# *Streptococcus pneumoniae* Serotype 12F-CC4846 and Invasive Pneumococcal Disease after Introduction of 13-Valent Pneumococcal Conjugate Vaccine, Japan, 2015–2017

**DOI:** 10.3201/eid2611.200087

**Published:** 2020-11

**Authors:** Satoshi Nakano, Takao Fujisawa, Yutaka Ito, Bin Chang, Yasufumi Matsumura, Masaki Yamamoto, Shigeru Suga, Makoto Ohnishi, Miki Nagao

**Affiliations:** Kyoto University Graduate School of Medicine, Kyoto, Japan (S. Nakano, Y. Matsumura, M. Yamamoto, M. Nagao);; National Hospital Organization Mie National Hospital, Tsu, Japan (T. Fujisawa, S. Suga);; Nagoya City University Graduate School of Medical Science, Nagoya, Japan (Y. Ito);; National Institute of Infectious Diseases, Tokyo, Japan (B. Chang, M. Ohnishi)

**Keywords:** *Streptococcus pneumoniae*, 12F, ST4846, 13-valent pneumococcal conjugate vaccine, PCV13, Japan, Tn6002, whole-genome sequence, bacteria, invasive pneumococcal disease

## Abstract

To prevent invasive pneumococcal disease (IPD), pneumococcal conjugate vaccines (PCVs) have been implemented in many countries; however, many cases of IPD still occur and can be attributable to nonvaccine serotypes of *Streptococcus pneumoniae*. In Japan, the number of IPD cases attributable to serotype 12F increased from 4.4% in 2015 to 24.6% in 2017 after 13-valent PCV was introduced. To clarify the associated genetic characteristics, we conducted whole-genome sequencing of 75 serotype 12F isolates. We identified 2 sequence types (STs) among the isolates: ST4846, which was the major type, and ST6945. Bayesian analysis suggested that these types diverged in »1942. Among serotype 12F-ST4846, we identified a major cluster, PC-JP12F, whose time of most recent common ancestor was estimated to be »2012. A phylogeographic analysis demonstrated that PC-JP12F isolates spread from the Kanto region, the most populated region in Japan, to other local regions.

*Streptococcus pneumoniae* is a common bacterial pathogen of children (*1*). To prevent pneumococcal infectious diseases, many countries have introduced 7-, 10-, and 13-valent pneumococcal conjugate vaccines (PCVs) (*2*), which have decreased the total number of invasive pneumococcal disease (IPD) cases globally. However, serotype shifts (i.e., increased identification of serotypes not in the PCV), were observed in areas in which PCVs were introduced (*3*–*6*); as a result, *S. pneumoniae* remains a major cause of bacterial infections, such as meningitis, pneumonia, and otitis media. In February 2010, PCV7 was licensed in Japan and was used on a voluntary basis until April 2013. During this period, the estimated rates of PCV7 vaccination for children <5 years of age increased from <10% in 2010 to 80%–90% in 2012 (*7*). In April 2013, use of PCV7 as a routine vaccine in Japan was approved, and in October 2013, vaccine for routine use was switched to PCV13.

To monitor the prevalence of different serotypes, sequence types (STs), and antimicrobial susceptibilities, we conducted a nationwide surveillance study of IPD and non-IPD cases in children in Japan during 2012–2017 (*8*,*9*). This passive nationwide surveillance was conducted by 254 medical institutions in Japan. During the first 3 years, 2012–2014, we detected decreased cases of PCV7 and PCV13 serotype pneumococcal infections and increased cases of serotype 24F IPD (*8*). During the next 3 years, 2015–2017, we detected markedly increased cases of serotype 12F IPD; 7 (4.4%) of 161 IPD isolates in 2015, 23 (13.9%) of 166 IPD isolates in 2016, and 42 (24.6%) of 171 IPD isolates in 2017 were classified as serotype 12F, although only 1 isolate classified as serotype 12F was detected during 2012–2014. Consequently, serotype 12F became the most prevalent serotype isolated from patients with IPD in 2017 in Japan (*9*). The mean age of these 73 IPD patients was 40.9 (range 3–126) months. Throughout the period, only 3 non-IPD serotype 12F isolates were detected out of a total of 231 non-IPD isolates. To clarify ST prevalence, penicillin-binding protein (PBP) profiles, resistance genes, and pili detection, we conducted whole-genome sequencing analysis of the serotype 12F isolates recovered in Japan. In addition, we used Bayesian-based phylogenetic analysis to investigate the dynamics of the spread.

## Materials and Methods

### Bacterial Isolates

From 23 of 47 prefectures in Japan, we obtained 1 serotype 12F IPD isolate in 2013 and 72 IPD and 3 non-IPD isolates during 2015–2017. Of the 76 isolates, 1 did not grow from the stock medium; we thus analyzed all 75 remaining isolates. We tested the antimicrobial susceptibility of the isolates to penicillin, cefotaxime, meropenem, erythromycin, and levofloxacin by using the broth microdilution method according to the Clinical and Laboratory Standards Institute guidelines (*10*). We used the MIC interpretive criteria for meningitis for this study.

### Basic Whole-Genome Sequencing Protocol

We extracted total genomic DNA and prepared sequence libraries by using a QIAamp DNA Mini Kit (QIAGEN, https://www.qiagen.com) and a Nextera XT DNA Library Preparation Kit (Illumina, https://www.illumina.com). We multiplexed and sequenced the samples by using an Illumina NextSeq system for 300 cycles (2 × 150-bp paired-end). The resulting sequences were assembled by using SPAdes version 3.13.1 (*11*). Mapping was performed by using Burrows-Wheeler Aligner version 0.7.17 (*12*) with *S. pneumoniae* strain ASP0581 (serotype 12F-ST4846, National Center for Biotechnology Information reference sequence NZ_AP019192.2) (*13*). Isolates with a mapping depth <20.0 were excluded from subsequent analysis. Multilocus sequence typing was performed by extracting all alleles from the assembled contigs by using BLAST+ version 2.6.0 (*14*) and a reference sequence of *S. pneumoniae* G54 (GenBank accession no. NC_011072.1). A clonal complex was defined as a group of STs sharing 5 of 7 loci in the multilocus sequence typing results.

### PBP Typing, Antimicrobial Resistance Genes, Pilus Detection, and Global Pneumococcal Sequence Cluster Identification

We assigned PBP transpeptidase domain type numbers to the extracted pbp*1a*, *2b*, and *2x* transpeptidase domain sequences of the examined isolates. The type numbers originated from previously published US Centers for Disease Control and Prevention PBP types (*15*–*18*). PBP types that had not been previously published in the US Centers for Disease Control and Prevention database were labeled with the prefix JP (e.g., *pbp1a*-JP1). Some of these original PBP types from Japan had been previously published (*19*–*21*). We detected *ermB*, *ermTR*, *mefA*, *mefE*, *tetM*, *tetO*, *rrgA-1* (pili1), and *pitB-1* (pili2) genes and searched for mutations and insertions/deletions within the *folA* and *folP* genes in the assembled contigs by following the standards published in a previous study (*15*). In addition, we assigned Global Pneumococcal Sequence Cluster (GPSC) numbers (*22*) and detected *tet*(S/M) by using Pathogenwatch (https://pathogen.watch).

### *Tn*916-like Integrative and Conjugative Element Analysis and *cps* Locus Comparison

We extracted the sequences of *Tn*916-like integrative and conjugative elements (ICEs) from the assembled contigs by using BLAST+ (https://blast.ncbi.nlm.nih.gov/Blast.cgi) and the *Enterococcus faecalis Tn*916 reference sequence (GenBank accession no. U09422.1). The analyzed sequences were annotated by using Prokka version 1.13.7 (*23*), and the structures of the regions were analyzed manually by using the Artemis Comparison Tool (*24*). In addition, we created a phylogenetic tree for the Tn916 region by using RAxML-ng version 0.9.0 (*25*). To obtain a phylogenetic tree of the *cps* locus, we mapped the trimmed reads to the serotype 12F *cps* locus reference sequence (GenBank accession no. CR931660.1) and obtained a phylogenetic tree by using RAxML (*26*).

### Recombination Site Detection and Phylogenetic Tree Construction

We constructed a phylogenetic tree by using Genealogies Unbiased By recomBinations In Nucleotide Sequences (Gubbins) version 2.2.1 (*27*). We mapped the obtained short reads to a the complete *S. pneumoniae* ASP0581 reference sequence (GenBank accession no. NZ_AP019192.2) (*13*) and input the aligned sequences into Gubbins, which identifies recombination events by using an algorithm that iteratively identifies loci containing increased densities of base substitutions while concurrently constructing a phylogeny based on the putative point mutations outside of these regions.

### Core-Genome Analysis

To clarify the differences in the genomic contents of the various clades, we used Prokka version 1.13.7 (*23*), Roary version 3.12.0 (*28*), and Scoary version 1.6.16 (*29*) to perform core-genome analysis. We defined genes that were exclusively found in a cluster at p<0.01, obtained with the Fisher exact test followed by the Bonferroni correction, as being specific to the cluster.

### Bayesian Analysis

We reconstructed a tree and obtained dates of the ancestors or nodes of the ST4846 and ST6945 clades by using the Bayesian Markov chain Monte Carlo framework. For this analysis, we performed recombination predictions by using the same protocol as described for all serotype 12F isolates. Final single-nucleotide polymorphism (SNP) alignments without recombination regions were used as the input dataset for BEAST version 1.10.4 (*30*). For the phylogeographic analysis, we used BEAUti (*30*) to additionally specify a symmetric discrete trait phylogeographic model by using a Bayesian stochastic search variable selection framework (*31*) as a metric for comparing geographic signals between datasets. We calculated Bayes factors indicating the transmission support by using SpreadD3 version 0.9.6 (*32*); consistent with convention, support was defined as a Bayes factor >3 (Appendix).

## Results

### STs and Antimicrobial Susceptibilities

Among the serotype 12F isolates recovered in Japan, we identified 2 STs: ST4846 (n = 59), which was the major ST, and ST6945 (n = 16), which was a double-locus variant of ST4846. Penicillin MICs for all serotype 12F isolates were <0.25 μg/mL. Of 59 ST4846 isolates, penicillin MICs for 16 isolates were <0.06 (susceptible), for 42 were <0.12 (resistant), and for 1 was <0.25 (resistant) (Table). Penicillin MICs for all but 1 of the 16 ST6945 isolates were <0.06 (susceptible). Of the 74 serotype 12F isolates, cefotaxime MICs for 69 isolates were <0.06 (susceptible), and meropenem MICs for 74 isolates were <0.06 (susceptible). For most isolates, erythromycin MICs were >128 (resistant, 71/74 isolates) and levofloxacin MICs were <1 (susceptible, 74/74 isolates).

**Table:** Antimicrobial susceptibilities of *Streptococcus pneumoniae* serotype 12F isolates recovered in Japan, 2017*

Sequence type	No. isolates	MIC, μg/mL														
		Penicillin				Cefotaxime				Meropenem		Erythromycin			Levofloxacin	
		<0.06	0.12	0.25		<0.06	0.12	0.25		<0.06		<0.06	>128		0.5	1
4846	59	16	42	1		54	3	2		59		0	59		4	55
6945	16	15	1	0		16	0	0		16		3	13		0	16

*Susceptibility categories were based on Clinical and Laboratory Standard 2015 antimicrobial susceptibility testing standards for *S. pneumoniae* (*10*). If categories for meningitis are available, they are shown. The standards are penicillin <0.06 susceptible, >0.12 resistant; cefotaxime <0.5 susceptible, 1.0 intermediate, >2 resistant; meropenem <0.25 susceptible, 0.5 intermediate, >1.0 resistant; erythromycin <0.25 susceptible, 0.5 intermediate, >1.0 resistant; levofloxacin <2.0 susceptible, 4.0 intermediate, >8.0 resistant.

### Whole-Genome Sequencing Statistics

The average (± SD) number of contigs was 55.8 (± 15.6), N50 (shortest contig length needed to cover 50% of the genome) was 69,627 (± 12,462), and mapping depth was 106.1 (± 36.1) (Appendix Tables 1, 2). One isolate had a mapping depth of 17.5 and was therefore excluded from the study.

### PBP Typing, Antimicrobial Resistance Genes, and Pilus Detection

**Figure 1:**
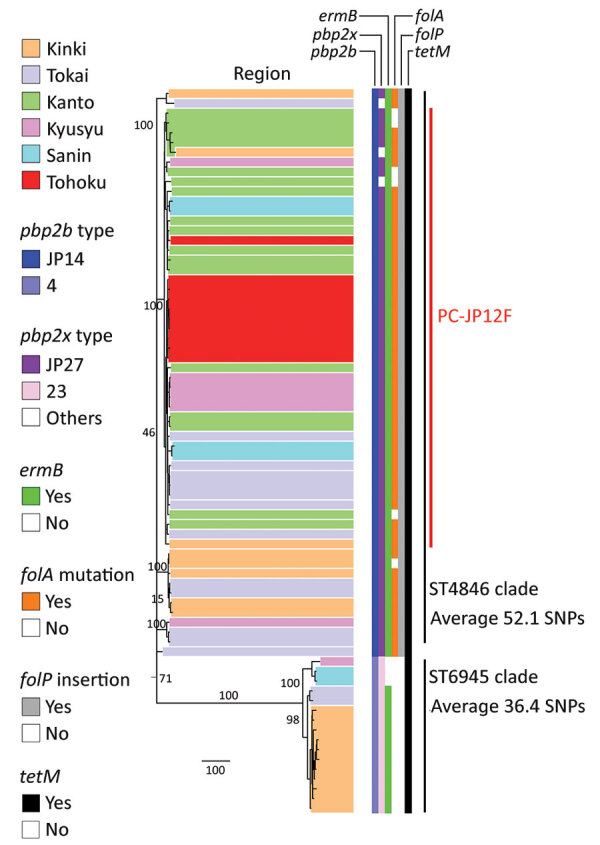
Recombination-free maximum-likelihood tree of *Streptococcus pneumoniae* serotype 12F-CC4846 isolates in Japan, created by using Gubbins (*27*). Two clusters were generated: 1 comprised only sequence type (ST) 4846 isolates and the other comprised only ST6945 isolates. All isolates had *pbp1a*-13. The *pbp2x* type “others” included *pbp2x*-JP23, *pbp2x*-JP58, and *pbp2x*-JP59. The geographic locations of the described regions in this figure are shown in the Appendix. The numbers on the branches indicate bootstrap values. SNP, single-nucleotide polymorphism; ST, sequence type.

All serotype 12F isolates contained *pbp1a*-37 (Figure 1; Appendix Table 1). All ST4846 isolates had *pbp2b*-JP14, and all ST6945 isolates contained *pbp2b-*4. We found 14 aa differences between *pbp2b*-JP14 and *pbp2b*-4. With regard to *pbp2x*, 55 of 58 ST4846 isolates had *pbp2x*-JP27 and all ST6945 isolates contained *pbp2x*-23. We found 19 aa differences between *pbp2x*-JP27 and *pbp2b*-23. In total, 55 of 58 ST4846 isolates had *pbp1a*:*pbp2b*:*pbp2x*, which equals 37:JP14:JP27, and all 16 ST6945 isolates contained *pbp1a*:*pbp2b*:*pbp2x*, which equals 37:4:23. All serotype 12F isolates had *tetM* and *ermB* with the exception of 3 ST6945 isolates that had only *tetM*. Of 58 ST4846 isolates, 52 carried the *folA* I100L mutation, but this mutation was not found in any of the ST6945 isolates. In addition, all ST4846 isolates had *folP* insertions, and none of the ST6945 isolates had mutations in this gene. None of the serotype 12F isolates carried *tetO*, *tet*(S/M), *ermTR*, *mefA*/*E*, Pili1, or Pili2. All serotype 12F isolates were assigned to GPSC334. Three isolates of GPSC334 were present in the GPSC database: 1 serotype 3 ST6945 isolate from Hong Kong and 2 serotype 12F isolates, ST1820 and ST1527, from Poland. Those 3 isolates were clustered into the ST6945 cluster in a subsequent recombination site-censored phylogenetic tree (Appendix Figure 1).

### *Tn*916-like ICE Structure and *cps* Locus Analysis

All serotype 12F isolates tested in this study had a *Tn*916-like ICE with *tetM*, which encodes tetracycline resistance. Of 58 ST4846 isolates, 57 had *Tn*6002 (*33*), which was found in a previous study to be one of the most common *Tn*916-like ICEs containing erythromycin-resistance cassettes between open reading frames 19 and 20 of *Tn*916 (*34*). We did not obtain a completely connected contig throughout the whole length of the *Tn*916-like ICE region for another ST4846 isolate. With regard to ST6945 isolates, we did not obtain completely connected contigs throughout the whole length of the *Tn*916-like ICE region. However, 13 of 16 ST6945 isolates had *ermB* insertions at the same position within the partial *Tn*916-like ICE as ST4846 isolates. The phylogenetic tree that was created by using the *cps* locus sequences generated a ST6945-specific clade that included all ST6945 isolates (Appendix Figure 2).

### Recombination Site Prediction, Phylogenetic Tree Construction, and Bayesian Analysis

**Figure 2:**
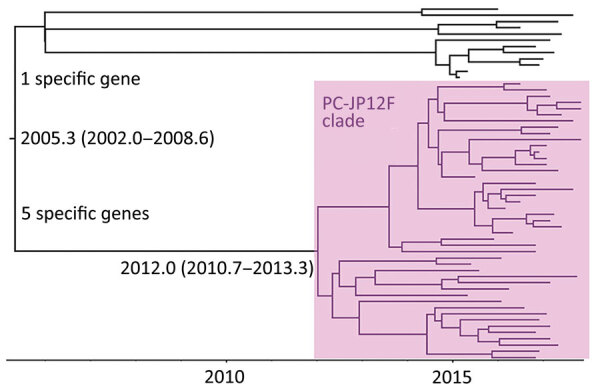
Maximum-clade credibility tree of *Streptococcus pneumoniae* sequence type (ST) 4846 clade isolates. The times of the most recent common ancestor are shown on the tree with 95% highest posterior density. This clade appeared to diversify in »2005, and the tree included 1 major clade, the PC-JP12F clade (pink shading), whose time of most recent common ancestor was »2012.0.

**Figure 3:**
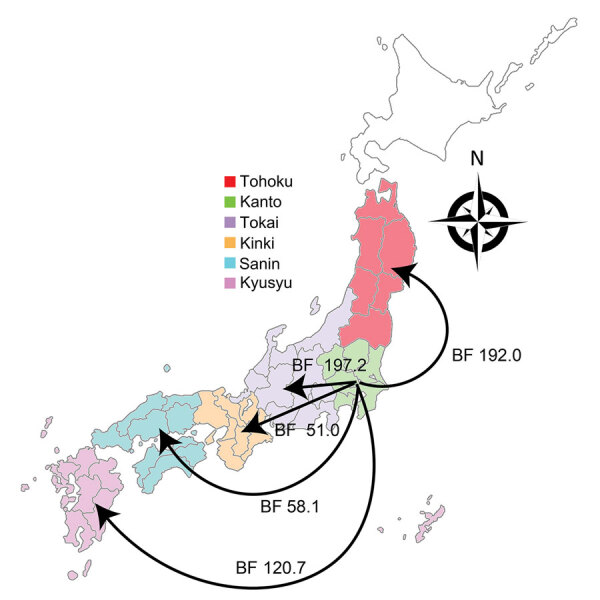
Bayesian phylogeography of *Streptococcus pneumoniae* serotype 12F isolates in the PC-JP12F clade between 6 discrete regions in Japan after the PC-JP12F clade arose. BFs indicate the transmission support; consistent with convention, support was defined as BF>3. Arrows indicate transmission direction. BF, Bayes factors.

When we constructed a recombination site censored phylogenetic tree of all serotype 12F isolates after recombination site prediction by using Gubbins (Figure 1; Appendix Figure 3), the tree identified 2 clusters composed exclusively of the ST4846 and ST6945 isolates. The average value of the pairwise SNP distances between isolates of the 2 clusters was 391, and the *r/m* value (average recombination to mutation rate) of the ST4846 clade was 0.3213 and of the ST6945 clade was 0.9639. One of the recombination sites overlapped with part of the nucleotide sequence of *pbp2b* (Appendix Figure 4). In addition, another recombination site overlapped with part of the *pbp2x* nucleotide sequence (Appendix Figure 4). No recombination site was found in the *cps* locus. We then performed a Bayesian analysis to estimate the time of most recent common ancestor (TMRCA) of the serotype 12F-CC4846 isolates by using BEAST. This analysis showed that serotype 12F-CC4846 in Japan arose in »1942 (95% highest posterior density [HPD] 1914–1963) (Appendix Figures 5, 6). In addition, 71 genes were unique to ST4846 isolates and 71 other genes were unique to ST6945 isolates. Although 16 of the 71 genes that were unique to ST6945 isolates did not exist in *S. pneumoniae* ASP0581, none of the 142 gene regions overlapped with the recombination regions predicted by Gubbins. We next used BEAST to estimate the TMRCA of the serotype 12F-ST4846 isolates based on only those belonging to the ST4846 clade. This analysis suggested that a common ancestor for our serotype 12F-ST4846 isolates arose in »2005 (95% HPD 2002–2009) (Figure 2; Appendix Figure) and generated 2 clades; the major clade (n = 44; 2015, 5/7 isolates; 2016, 17/23 isolates; 2017, 23/42 isolates), PC-JP12F, which arose in »2012 (95% HPD 2011–2013), had 5 genes that were lacking in the other ST4846 isolates (Appendix Table 3). This result indicated that the isolates included in the PC-JP12F clade spread rapidly in Japan; therefore, we conducted a phylogeographic analysis of the isolates to clarify the transmission over time. This analysis revealed 5 statistically supported (Bayes factor >3) routes of transmission between 6 discrete regions in Japan (Figure 3). All of the supported transmission routes originated from the Kanto region, which is the central populated region of Japan, and spread to all 5 other local regions. The highest support was obtained for transmission from the Kanto region to the Tokai region (Bayes factor 197.2), which is contiguous to the Kanto region.

The TMRCA of serotype 12F-ST6945 isolates, estimated by using only ST6945 isolates, was 1997 (95% HPD 1925–2005; Appendix Figure 8). The phylogenetic tree of the *Tn*916-like ICE region generated ST6945-specific and PC-JP12F–specific clades (Appendix Figure 9). In addition, all *ermB-*negative ST6945 isolates created a subclade within the ST6945-specific clade.

## Discussion

In our nationwide surveillance study of pneumococcal disease in children, conducted in Japan during 2012–2017, we detected a rapid increase in serotype 12F IPD. Thus, we performed a whole-genome sequencing–based molecular analysis to clarify the associated genomic characteristics and their dynamics. We found 2 lineage STs within the serotype 12F isolates recovered in Japan: ST4846, which was the major sequence type, and ST6945, which was a double-locus variant of ST4846. To investigate whether these 2 STs had the same ancestor, we compared their genetic characteristics by whole-genome sequencing. According to the STs and the finding that both ST4846 and ST6945 isolates belonged to GPSC334, these isolates appear to be closely related. Although this GPSC334 was a minor cluster in the original study and the isolates from East Asia used in the study were limited (*22*), the average pairwise SNP distance between the 2 ST isolates was 391; this value is reasonable based on the evidence that the ST4846 and ST6945 isolates belonged to the same sequence cluster in the original study. However, we found several genetic differences between the 2 lineage STs, such as differences in the PBP profile, the prevalence of *folA* mutations and *folP* insertions, the *Tn*916 structure, and the *cps* locus sequences. In addition, we found 142 genetic differences between the 2 STs in the core-genome analysis. In general, *S. pneumoniae* is a paradigm for recombination, and in our study, we certainly identified recombination sites, particularly in the ST6945 cluster. Therefore, we believe that these recombination events caused the discrepancy after its divergence in »1942. Of note, recombination sites were not identified in the *cps* locus although the phylogenetic tree for the *cps* locus generated ST-specific clades. Considering that the process of evolving from a common ancestor to 2 distinctive clades is a result of randomly accumulated mutations, recombination events, or both, there might be ST-specific genetic backgrounds that influenced the dynamics of the *cps* region.

We found a major clade within the ST4846 isolates (i.e., PC-JP12F clade) that seemed to spread rapidly in Japan. Bayesian analysis suggested that this clade arose in »2012. This estimation showed a narrow 95% HPD, and we therefore believe that this estimated date was reliable. Thus, the rapid spread and high prevalence of serotype 12F-CC4846 in Japan appeared be mainly caused by this strain. In addition, the phylogeographic analysis suggested the route of transmission of this strain, which mainly involved spread from the Kanto region to other local regions. The Kanto region has 7 prefectures, including Tokyo, the capital of Japan, which contains »35% of the population of Japan and is thus the most populated of all regions in Japan. In general, in the phylogeographic analysis, Bayes factors >100 indicate decisive, 30–100 indicate very strong, 10–30 indicate strong, and 3–10 indicate substantial support for a model (*35*). Therefore, we believe that the determined transmission routes in Japan (i.e., from the urban region to countryside regions) were reasonable and reliable.

To date, 3 studies have demonstrated outbreaks of serotype 12F-CC4846 in Japan during 2016–2018 (*36*–*38*). Of the 3 outbreaks, 2 can be attributed to ST4846 isolates that occurred in the Chiba Prefecture in the Kanto region and in the Yamagata Prefecture in the Tohoku region. The other outbreak was attributable to ST6945 isolates in the Hyogo Prefecture in the Kinki region; in our study, ST6945 isolates were also recovered in the Kinki region (Figure 1). In addition, Shimbashi et al. reported that although the data were obtained from a pneumococcal surveillance study among adults, the ST6945 isolates were recovered in the Tokai, Kyusyu, and Tohoku regions during 2015–2017 (*39*). Given these findings, the serotype 12F-ST4846 isolates had already spread throughout Japan, and the serotype 12F-ST6945 isolates were still limited to several small regions but had already started spreading in Japan.

Serotype 12F isolates were recovered mainly from patients with IPD, including outbreak cases, in many areas globally after the introduction of PCV13 (*40*–*43*); several studies have demonstrated that serotype 12F is associated with high morbidity and mortality rates (*37*,*44*). The STs of serotype 12F isolates in different countries exhibit differences, which indicates that prevalence of these serotype 12F isolates was not the result of global but rather of regional clonal spread. In addition, Gladstone et al. and Balsells et al. found invasiveness to be relatively higher for serotype 12F pneumococci than for other serotypes (*22*,*45*). Of note, according to the GPSC database (*22*), all serotype 12F isolates are susceptible or mildly resistant to penicillin (MIC <0.12), similar to the results obtained for the isolates included in our study. Therefore, it is unlikely that antibiotic pressure caused the spread in Japan and other regions. Considering these findings, serotype 12F should exhibit high invasiveness and probably has the potential to be transmitted efficiently and thus cause an outbreak or regional spread. With regard to this issue, the mechanism underlying the rapid spread and high invasiveness of serotype 12F strains should be determined in future studies.

In this study, we identified the structures of *Tn*916-like ICEs in serotype 12F-CC4846. Most serotype 12F-ST4846 isolates contained *Tn*6002, which was also widely detected in serotype 15A-CC63 isolates in Japan (*19*,*20*). The phylogenetic tree of the *Tn*916-like ICE region suggested that the origins of the *Tn*916-ICE region in PC-JP12F isolates might differ from those of the other serotype 12F-CC4846 isolates. However, we should note that the genetic difference might be caused by mapping errors (i.e., the choice of reference sequence). In Japan, the macrolide resistance rate was >90% (*9*); thus, further studies on *Tn*916-like ICEs, including its epidemiology, transmission mechanism, and functions that influence the dynamics of pneumococci, are needed.

We note some limitations of this study. First, we tested pneumococcal isolates that were collected in a nationwide surveillance study during 2012–2017. However, all isolates were recovered during 2015–2017, except for 1 that was recovered in 2013. Therefore, this short sampling period might affect the molecular clock analysis (i.e., TMRCA estimation). The TMRCAs of the serotype 12F-CC4846 isolates (Appendix Figure 5) and ST6945 (Appendix Figure) had very broad 95% HPDs. Therefore, we believe that longer samplings are needed to more accurately estimate the dates. In contrast, the TMRCA of ST4846 was estimated with a narrow 95% HPD. Thus, we believe that the results of the date estimations and the subsequent analyses (Figure 2) were robust and reliable.

In conclusion, we found rapid spread of serotype 12F-CC4846 isolates among patients with IPD in Japan after the introduction of PCV13. The results identified ST4846 and ST6945 (double-locus variant of ST4846) lineages for the serotype 12F-CC4846 isolates in Japan, but many genetic differences were found between the 2 lineages. Bayesian analysis identified a major cluster within the ST4846 isolates (PC-JP12F cluster). This PC-JP12F cluster arose in »2012 and rapidly spread from the Kanto region to countryside regions. As we showed in this study, *S. pneumoniae* serotype 12F lineages could have the potential to spread rapidly; therefore, we should monitor the trend of the lineages when they are detected.

Appendix Supplementary methods and results for study of *Streptococcus pneumoniae* serotype 12F-CC4846 and invasive pneumococcal disease after introduction of 13-valent pneumococcal conjugate vaccines, Japan, 2017.
