# Supplementary material for: Streptococcus pneumoniae Serotype 12F-CC4846 and Invasive Pneumococcal Disease after Introduction of 13-Valent Pneumococcal Conjugate Vaccine, Japan, 2015–2017
Source: Emerg Infect Dis. 2020 Nov;26(11):2660–8. doi: 10.3201/eid2611.200087 (PMC7588537; doi:10.3201/eid2611.200087)
Supplement: Appendix — Supplementary methods and results for study of Streptococcus pneumoniae serotype 12F-CC4846 and invasive pneumococcal disease after introduction of 13-valent pneumococcal conjugate vaccines, Japan, 2017. [file 20-0087-Techapp-s1.pdf]

Article DOI: https://doi.org/10.3201/eid2611.200087

Streptococcus pneumoniae Serotype 12F-CC4846 and Invasive Pneumococcal Disease after Introduction of 13-Valent Pneumococcal Conjugate Vaccine, Japan, 2017

Appendix

Supplementary Methods and Results

We obtained draft genome data of the serotype 12F isolates that were recovered in Japan between 2013 and 2017. We identified the STs, resistance genes, pili and *pbp1a*, *2b* and *2x* sequences based on assembled contigs using BLAST+ v.2.6.0 (*1*). For MLST, we downloaded reference sequences of each allele from pubMLST (https://pubmlst.org/). We then compared the reference sequences to contigs; extracted sequences that were completely identical to the reference sequence throughout the region were adopted as the allelic numbers. With regard to the resistance genes *pili1* and *pili2*, reference sequences were obtained using BLAST analysis in a previous study (*2*). The reference sequences of all PBPs were obtained from the *S. pneumoniae* G54 reference sequence that was submitted as NC_011072.1 to the GenBank database. PBP types were assigned using a previously published PBP database (*2*), and the Japanese original types were published elsewhere (*3*–*5*). The novel PBP profile identified in Japan is described below.

Recombination site prediction and phylogenetic analysis

We utilized a single-nucleotide polymorphism (SNP)-based approach to create a phylogenetic tree using Gubbins v.2.2.1 (*6*). After trimming the obtained raw reads using fastp v.0.20.0 (*7*), the reads were mapped to the *S. pneumoniae* ASP0581 complete sequence (GenBank accession No. NZ_AP019192.2) using the Burrows-Wheeler Aligner (*8*) because the isolate was closely related to the tested serotype 12F isolates. After the removal of duplicate

reads and indels using the GATK Best Practices workflow (*9*), consensus sequence FASTA files were created using VCFtools (*10*). Low-quality mapping reads with the parameters DP>5, MQ>30 and QD>20 were removed using vcffilter. The Gubbins analysis was performed using the standard parameters. The average recombination rate (*r/m*) was calculated from the data obtained with Gubbins. Ancestral recombination events that occurred once and spread in the cluster via clonal descent were counted once.

For establishment of the *cps* locus-based phylogenetic tree, we mapped the trimmed reads to the serotype 12F *cps* locus reference sequence (GenBank accession No. CR931660.1), and a phylogenetic tree was obtained using RAxML v8.2.10 (*11*) with the GTRGAMMA DNA substitution model. The node support was assessed using 500 bootstrap replicates. The model decision was based on the ModelTest-NG results.

*Tn*916 analysis

We extracted the sequences of *Tn*916-like ICEs from the assembled contigs using BLAST+. The reference sequence of *Tn*916 (GenBank accession No. U09422.1) was used for the extraction. The analyzed sequences were annotated using Prokka v1.13.7 (*12*) with the standard parameters. The detailed structures of the region were visualized using ACT (*13*) and assigned their transposon types. In addition, we conducted a phylogenetic analysis using all serotype 12F isolates. The trimmed reads were mapped on the reference *Tn*916 sequence, and the alignment was analyzed using RAxML-ng (*14*) with GTR+G4. The reference sequence of *Tn*916 was used for the outgroup. The model decision was based on the ModelTest-NG results, and the node support was assessed using 3000 bootstrap replicates.

Core genome analysis

We annotated the assembled contigs using Prokka with the standard parameters. After the annotation, we searched pan and core genomes using Roary v3.12.0 (*15*) with the standard parameters. For all serotype 12F-CC4846 isolates, we identified a total of 2346 genes, including 1898 core genes that were shared by more than 99% of the isolates. For the serotype 12F-ST4846 isolates, we identified a total of 2235 isolates, including 1976 core genes.

Within the target clusters, we searched specific genes of the cluster using Scoary v1.6.16 with the standard parameters, and genes that were exclusive found in each cluster with a *p* value

<0.01 obtained with Fisher's exact tests followed by the Bonferroni correction were defined as specific genes of the cluster.

The gene regions identified to be ST4846- or ST6945-specific were compared with recombination regions in the Gubbins analysis. We identified the gene regions in the *S. pneumoniae* ASP0581 complete sequence by BLAST+ searching using the gene sequences. Then, we compared the regions with the recombination regions predicted by Gubbins.

Bayesian analysis

We estimated the time of the most recent common ancestor (TMRCA) of the ST4846 and ST6945 clades using BEAST v.1.10.4. The input file for the BEAST analysis did not include recombination regions, and SNP alignment files for each isolate were obtained. For examination of the temporal signal of the inputted dataset, we used TempEst (*16*) to diagnose the regression of the root-to-tip genetic distance against the sampling time. Analysis of the best-fitting root mean square of the heuristic residual showed that the correlation coefficients (R^2) of all serotype 12F isolates, ST4846 clade isolates, and ST6945 isolates were 0.3405, 0.4194 and $5.8502\times E^{-2}$, respectively. Although the R^2 of the ST6945 isolates was low, we did not remove any sample because we decided that this quality was due to the short sampling period and/or the small number of isolates belonging to the ST6945 clade. The model was selected through comparisons of the marginal likelihood using path sampling and stepping stone-based marginal likelihood estimation for a strict clock and an uncorrelated relaxed clock in a molecular clock model and a constant size and Bayesian Skygrid in a tree prior model. Consequently, we selected a strict-clock model and a Bayesian Skygrid model for the ST4846 clade analysis and strict clock model and a Bayesian Skygrid model for the ST6945 clade analysis. To obtain an effective sample size (ESS) greater than 200 for all factors, we set the MCMC lengths for ST4846 and ST6945 to 2.0×10^8 and 1.0×10^9, respectively. We specified a general-time-reversible substitution model with site rate heterogeneity modeled across four gamma distributions (GTR+Γ4) for all analyses.

We found one major subclade within the ST4846 clade in the maximum clade credibility tree; thus, we performed a phylogeographic analysis of the 45 ST4846 isolates. We specified an asymmetric discrete trait phylogeographic model utilizing a Bayesian stochastic search variable selection (BSSVS) framework (*17*) as a metric for comparing the geographic signals between

datasets. For computational efficiency, we aggregated the 14 discrete sampling prefectures into six geographical regions (which are usually used in Japan) in the asymmetric model.

Japan original PBP profile

pbp1a

>JP_1a1

SMKPITDYAPALEYGVYDSTASIVHDVPYNYPGTDTPLYNWDHVYFGNITIQYA
LQQSRNVTAVETLNKVGLDRAKTFLNGLGIDYPSMHYANAISSNTTESNKKYGASSEK
MAAAYAAFANGGIYHKPMYINKIVFSDGSEKEFSDAGTRAMKETTAYMMTEMMKTVL
TYGTGRGAYLPWLPQAGKTGTSNYTDEEIEKYIKNTGYVAPDEMFVGYTRKYSMAVW
TGYSNRLTPIVGDGFLVAAKVYRSMITYLSEDTHPEDWTMPDGLFRNGEFV

>JP_1a2

SMKPITDYAPALEYGVYDSTATIVHDEPYNYPGTDIPVYNWDRGYFGNITLQYA
LQQSRNVPAVETLNKVGLNRAKTFLNGLGIDYPSLHYSNAISSNTTESDQKYGASSEKM
AAAYAAFANGGTYYKPMYIHKVVFSDGSEKEFSNVGTRAMKETTAYMMTDMMKTVL
TYGTGRGAYLPWLPQAGKTGTSNYTDEEIEKYIKNTGYVAPDEMFVGYTRKYSMAVW
TGYSNRLTPLVGNGLTVAAKVYRSMMTYLSEGSNPEDWNIPEGLYRNGEFV

>JP_1a3

SMKPITDYAPALEYGVYDSTASIVHDVPYNYPGTDTPLYNWDHVYFGNITIQYA
LQQSRNVTAVETLNKVGLDRAKTFLNGLGIDYPSMHYANAISSNTTESNKKYGASSEK
MAAAYAAFANGGIYHKPMYINKIVFSDGSEKEFSDAGTRAMKETTAYMMTDMMKTVL
SYGTGRNAYLAWLPQAGKTGTSNYTDEEIEKYIKNTGYVAPDEMFVGYTRKYSMAVW
TGYSNRLTPIVGDGFLVAAKVYRSMMTYLSEGSNPEDWNIPEGLYRNGEFV

>JP_1a4

TMKPITDYAPAIEYGIYDSTATMVNDIPYNYPGTSTPVYNWDRAYFGNITLQYAL
QQSRNVPAVETLNKVGLDRAKNFLNGLGIDYPDMHYSNAISSNTTESNKQYGASSEKM
AAAFAAFANGGIYHKPMYINKIVFSDGSEKEFSDAGTRAMKETTAYMMTEMMKTVLS
YGTGRNAYLAWLPQAGKTGTSNYTDEEIENHIKTSQFVAPDELFAGYTRKYSMAVWTG
YSNRLTPLVGNGLTVAAKVYRSMMTYLSEGSNPEDWNIPEGLYRNGEFV

>JP_1a5

TMKPITDYAPAIEYGVYDSTATMVNDIPYNYPGTSTPVYNWDRAYFGNITLQYA
LQQSRNVPAVETLNKVGLDRAKNFLNGLGIDYPDMHYSNAISSNTTESNKQYGASSEK
MAAAFAAFANGGIYHKPMYINKIVFSDGSEKEFSDAGTRAMKETTAYMMTEMMKTVL
SYGTGRNAYLAWLPQAGKTGTSNYTDEEIENHIKTSQFVAPDELFVGYTRKYSMAVWT
GYSNRLTPLVGNGLTVAAKVYRSMMTYLSEGSNPEDWNIPEGLYRNGEFV

>JP_1a6

SMKPITDYAPALEYGVYDSTASIVHDVPYNYPGTDTPLYNWDHVYFGNITIQYA
LQQSRNVTAVETLNKVGLDRAKTFLNGLGIDYPSMHYANAISSNTTESNKKYGASSEK
MAAAYAAFANGGIYHKPMYIHKVVFSDGSEKEFSDAGTRAMKETTAYMMTEMMKTV
LTYGTGRGAYLPWLPQAGKTGTSNYTDEEIEKYIKNTGYVAPDEMFVGYTRKYSMAV
WTGYSNRLTPIVGDGFLVAAKVYRSMMTYLSEGSNPEDWNIPEGLYRNGEFV

>JP_1a7

TMKPITDYAPAIEYGVYDSTATMVNDIPYNYPGTSTPVYNWDRAYFGNITLQYA
LQQSRNVPAVETLNKVGLDRAKNFLNGLGIDYPDMHYSNAISSNTTESNKQYGASSEK
MAAAFAAFANGGIYHKPMYINKIVFSDGSEKEFSDAGTRAMKETTAYMMTEMMKTVL
SYGTGRNAYLAWLPQAGKTGTSNYTDKEIENHIKTSQFVAPDELFAGYTRKYSMAVWT
GYSNRLTPLVGNGLTVAAKVYRSMMTYLSEGSNPEDWNIPEGLYRNGEFV

>JP_1a8

SMKPITDYAPALEYGVYDSTATMVNDIPYNYPGTSTPVYNWDRAYFGNISLQYA
LQQSRNVPAVETLNKVGLDRAKTFLNGLGIDYPSIHYSNAISSNTTESSKQYGASSEKMA
AAYAAFANGGIYHKPMYINKVVFSDGSEKEFSDAGTRAMKETTAYMMTEMMKTVLTY
GTGRGAYLPWLPQAGKTGTSNYTDDEIEKYVKNTGYVAPDEMFVGYTRKYSMAVWT
GYSNRLTPIIGDGFLVAAKVYRSMISYLSEDDHPGDWTMPEGVYRSGEFV

>JP_1a9

SMKPITDYAPALEYGVYDSTATMVNDIPYNYPGTSTPVYNWDRAYFGNISLQYA
LQQSRNVPAVETLNKVGLDRAKTFLNGLGIDYPSIHYSNAISSNTTESSKQYGASSEKMA
AAYAAFANGGIYHKPMYINKVVFSDGSEKEFSDTGTRAMKETTAYMMTEMMKTVLTY

GTGRGAYLPWLPQAGKTGTSNYTDDEIEKYIKNTGYVAPDEMFVGYTRKYSMAVWTG
YSNRLTPIIGDGFLVAAKVYRSMISYLSEDDHPGDWTMPEGVYRSGEFV

>JP_1a10

SMKPITDYAPALEYGVYDSTASIVHDVPYNYPGTDTPLYNWDHVYFGNITIQYA
LQQSRNVTAVETLNKVGLDKAKTFLNGLGIDYPSMHYANAISSNTTESNKKYGASSEK
MAAAYAAFANGGIYHKPMYINKIVFSDGSEKEFSDAGTRAMKETTAYMMTEMMKTVL
TYGTGRGAYLPWLPQAGKTGTSNYTDEEIEKYIKNTGYVAPDEMFVGYTRKYSMAVW
TGYSNRLTPIVGDGFLVAAKVYRSMITYLSEDTHPEDWTMPDGLFRNGEFV

>JP_1a11

TMKPITDYAPALEYGVYDSTATIVHDEPYNYPGTNTPVYNWDRGYFGNITLQYA
LQQSRNVPAVETLNKVGLNRAKTFLNGLGIDYPSIHYSNAISSNTTESDKKYGASSEKM
AAAYAAFANGGTYYKPMYIHKVVFSDGSEKEFSNVGTRAMKETTAYMMTDMMKTVL
SYGTGRNAYLAWLPQAGKTGTSNYTDEEIENHIKTSQFVAPDELFAGYTRKYSMAVWT
GYSNRLTPLVGNGLTVAAKVYRSMMTYLSEGGNPEDWNIPEGLYRNGEFV

>JP_1a12

TMKPITDYAPALEYGVYDSTATIVHDEPYNYPGTNTPVYNWDRGYFGNITLQYA
LQQSRNVPAVETLNKVGLNRAKTFLNGLGIDYPSIHYSNAISSNTTESDKKYGASSEKM
AAAYAAFANGGTYYKPMYIHKVVFSDGSEKEFSNVGTRAMKKTTAYMMTDMMKTVL
SYGTGRNAYLAWLPQAGKTGTSNYTDEEIENHIKTSQFVAPDELFAGYTRKYSMAVWT
GYSNRLTPLVGNGLTVAAKVYRSMMTYLSEGSNPEDWNIPEGLYRNGEFV

>JP_1a13

TMKPITDYAPALEYGVYDSTATIVHDEPYNYPGTNTPVYNWDRGYFGNITLQYA
LQQSRNVPAVETLNKVGLNRAKTFLNGLRIDYPSIHYSNAISSNTTESDKKYGASSEKM
AAAYAAFANGGTYYKPMYIHKVVFSDGSEKEFSNVGTRAMKETTAYMMTDMMKTVL
SYGTGRNAYLAWLPQAGKTGTSNYTDEEIENHIKTSQFVAPDELFAGYTRKYSMAVWT
GYSNRLTPLVGNGLTVAAKVYRSMMTYLSEGSNPEDWNIPEGLYRNGEFV

>JP_1a14

TMKPITDYAPALEYGVYDSTATIVHDEPYNYPGTNTPVYNWDRGYFGNITLQYA LQQSRNVPAVETLNKVGLNRAKTFLNGLGIDYPSIHYSNAISSNTTESDKKYGASSEKM AAAYAAFANGGTYYKPMYIHKVVFSDGSEKEFSNVGTRAMKETTTYMMTDMMKTVL TYGTGRNAYLAWLPQAGKTGTSNYTDEEIENHIKTSQFVAPDELFAGYTRKYSMAVWT GYSNRLTPLVGNGLTVAAKVYRSMMTYLSEGSNPEDWNIPEGLYRNGEFV

>JP_1a15

SMKPITDYAPALEYGVYDSTASIVHDVPYNYPGTDTPLYNWDHVYFGNITIQYA LQQSRNVTAVETLNKVGLDRAKTFLNGLGIDYPSMHYANAISSNTTESNKKYGASSEK MAAAYAAFANGGIYHKPMYINKIVFSDGSEKEFSDAGTRAMKETTAYMMTEMMKTVL TYGTGRGAYLPWLPQAGKTGTSNYTDEEIEKYIKNTGYVAPDEMFVGYTRKYSMAVW TGYSNRLTPIVGDGFLVAAKVYRSMISYLSEDDHPGDWTMPEGLYRSGEFV

>JP_1a16

SMKPITDYAPALEYGVYDSTASIVHDVPYNYPGTDTPLYNWDHVYFGNITIQYA LQQSRNVTAVETLNKVGLDRAKTFLNGIGIDYPDMHYANAISSNTTESNKKYGASSEKM AAAYAAFANGGIYHKPMYINKIVFSDGSSKEYADPGTRAMKETTAYMMTEMMKTVLA YGTGRGAYLPWLPQAGKTGTSNYTDDEIENYIKNTGYVAPDEMFVGYTRKYSMAVWT GYSNRLTPIVGDGFYVAAKVYRSMMTYLSEDNNPGDWTMPDGLFRNGEFV

>JP_1a17

SMKPITDYAPALEYGVYDSTATIVHDEPYNYPGTDIPVYNWDRGYFGNITLQYA LQQSRNVPAVETLNKVGLNRAKTFLNGLGIDYPSLHYSNAISSNTTESDQKYGASSEKM AAAYAAFANGGTYYKPMYIHKVVFSDGSEKEFSNVGTRAMKETTAYMMTDMMKTVL TYGTGRGAYLPWLPQAGKTGTSNYTDEEIEKYIKNTGYVAPDEMFVGYTRKYSMAVW TGYSNRLTPIIGDGFLVAAKVYRSMMTYLSEGSNPEDWNIPEGLYRNGEFV

>JP_1a18

SMKPITDYAPALEYGVYDSTATIVHDEPYNYPGTDIPVYNWDRGYFGNITLQYA LQQSRNVPAVETLNKVGLNRAKTFLNGLGIDYPSLHYSNAISSNTTESDQKYGASSEKM AAAYAAFANGGTYYKPMYIHKVVFSDGSEKEFSNVGTRAMKETTAYMMTDMMKTVL

TYGTGRGAYLPWLPQAGKTGTSNYTDEEIEKYIKNTGYVAPDEMFVGYTRKYSMAVW TGYSNRLTPIIGDGFLVAAKVYRSMMTYLSEGSNPEDWNMPDGIYRNGEFV

pbp2b

>JP_2b1

TNVFVPGSVVKAATISSGWENGVLSGNQTLTDQPIVFQGSAPIYSWYKLAYGSFP ITAVLEYSSNAYMVQTALGIMGQTYQPNMFVGTSNLESAMEKLRSTFGEYGLGTATGID LPDTGFVPKEYSFANYITNAFGQFDNYTPMQLAQYVATIANNGVRVAPRIVEGIYGNND KGGDLIQQLQPTEMNKVNISDSDMSILHQGFYQVAHGTSGLTTGRAFSNGAAVSISGKT GESYVEGGQEANNTNAVAYAPSDNPQIAVAVVFPHNTN

>JP_2b2

TNVFVPGSVVKAATISSGWENGVLSGNQTLTDQPIVFQGSAPIYSWYKLAYGSFP ITAVLEYSSNAYMVQTALGIMGQTYQPNMFVGTSNLETAMGKLRATFGEYGLGAATGI DLPDTGFVPKEYSFANFITNAFGQFDNYTPMQLAQYVATIANNGVRLAPHIVEGIYDNN DKGGELIQAIDTKEINKVNISESDMAILHQGFYQVSHGTSPLTTGRAFSDGATVSISGKTG ESYVAGGQEANNTNAVAYAPTENPQIAVAVVFPHNTN

>JP_2b3

TNVFAPGSVVKAATISSGWENGVLSGNQTLTDQSIVFQGSAPINSWYTQAYGSFP ITAVLEYSSNAYMVQTALGLMGQTYQPNMFVGTSNLESAMGKLRSTFGEYGLGSATGI DLPDTGFVPKDYSFANYITNAFGQFDNYTPMQLAQYVATIANDGVRVAPRIVEGIYGNN DKGGDLIQQLQPTEMNKVNISDSDMSILHQGFYQVAHGTSGLTTGRAFSNGAAVSISGK TGESYVEGGQEANNTNAVAYAPSDNPQIAVAVVFPHNTN

>JP_2b4

TNVFVPGSVVKAATISSGWENGVLSGNQTLTDQSIVFQGSAPINSWYTQAYGSFP ITAVLEYSSNAYMVQTALGLMGQTYQPNMFVGTSNLESAMGKLRSTFGEYGLGSATGI DLPDTGFVPKDYSFANYITNAFGQFDNYTPMQLAQYVATIANDGVRVAPRIVEGIYGNN DKGGDLIQQLQPTEMNKVNVSDSDMSILHQGFYQVAHGTSGLTTGRAFSNGAAVSISG KTGESYVEGGQEANNTNAVAYAPSDNPQIAVAVVFPHNTN

>JP_2b5

TNVFVPGSVVKAATISSGWENGVLSGNQTLTDQSIVFQGSAPINSWYTQAYGSFP ITAVLEYSSNVYMVQTALGLMGQTYQPNMFVGTSNLESAMGKLRSTFGEYGLGSATGI DLPDTGFVPKDYSFANYITNAFGQFDNYTPMQLAQYVATIANDGVRVAPRIVEGIYGNN DKGGDLIQQLQPTEMNKVNISDSDMSILHQGFYQVAHGTSGLTTGRAFSNGAAVSISGK TGESYVEGGQEANNTNAVAYAPSDNPQIAVAVVFPHNTN

>JP_2b6

TNVFVPGSVVKAATISSGWENGVLSGNQTLTDQSIVFQGSAPINSWYTQAYGSFP ITAVLEYSSNTYMVQTALGLMGQTYQPNMFVGTSNLESAMEKLRSTFGEYGFGTATGI DLPDIGFVPKEYSFANYITNAFGQFDNYTPMQLAQYVATIANNGVRVAPRIVEGIYGNN DKGGDLIQQLQPTEMNKVNISDSDMSILHQGFYQVAHGTSGLTTGRAFSNGALVSISGK TGESYVADGQQATNTNAVAYAPSDNPQIAVAVVFPHNTN

>JP_2b7

TNVFVPGSVVKAATISSGWENGVLSGNQTLTDQSIVFQGSAPINSWYTQAYGSFP ITAVLEYSSNTYMVQTALGLMGQTYQPNMFVGTSNLESAMEKLRSTFGEYGLGTATGI DLPDIGFVPKEYSFANYITNAFGQFDNYTPMQLAQYVATIANNGVRVAPRIVEGIYGNN DKGGDLIQQLQPTEMNKVNISDSDMSILHQGFYQVAHGTSGLTTGRAFSNGALVSISGK TGESYVADGQQATNTNAVAYAPSDNPQIAVAVVFPHNTN

>JP_2b8

TNVFVPGSVVKAATISSGWENGVLSGNQTLTDQPIVFQGSAPIYSWYKLAYGSFP ITAVLEYSSNAYMVQTALGIMGQTYQPNMFVGTSNLETAMGKLRATFGEYGFGAATGI DLPDTGFVPKEYSFANYITNAFGQFDNYTPMQLAQYVATIANDGVRVAPRIVEGIYGNN DKGGDLIQQLQPTEMNKVNISDSDMSILHQGFYQVAHGTSGLTTGRAFSNGALVSISGK TGESYVADGQQATNTNAVAYAPSDNPQIAVAVVFPHNTN

>JP_2b9

TNVFVPGSVVKAATISSGWENGVLSGNQTLTDQPIVFQGSAPIYSWYKLAYGSFP ITTVLEYSSNAYMVQTALGIMGQTYQPNMFVGTSNLETAMGKLRATFGEYGLGAATGI DLPDTGFVPKEYSFANYITNAFGQFDNYTPMQLAQYVATIANDGVRVAPRIVEGIYGNN

DKGGDLIQQLQPTEMNKVNISDSDMSILHQGFYQVAHGTSGLTTGRAFSNGALVSISGK
TGESYVAGGQEANNTNAVAYAPSDNPQIAVAVVFPHNTN

>JP_2b10

TNVFVPGSVVKAATISSGWENGVLSGNQTLTDQPIVFQGSAPIYSWYKLAYGSFP
ITAVLEYSSNAYMVQTALGIMGQTYQPNMFVGTSNLETAMGKLRATFGEYGLGAATGI
DLPDTGFVPKEYSFANYITNAFGQFDNYTPMQFAQYVATIANDGVRVAPRIVEGIYGNN
DKGGDLIQQLQPTEMNKVNISDSDMSILHQGFYQVAHGTSGLTTGRAFSNGALVSISGK
TGESYVAGGQEANNTNAVAYAPSDNPQIAVAVVFPHNTN

>JP_2b11

TNVFVPGSVVKAATISSGWENGVLSGNQTLTDQPIVFQGSAPIYSWYKLAYGSFP
ITAVLEYSSNAYMVQTALGIMGQTYQPNMFVGTSNLETAMGKLRATFGEYGLGAATGI
DLPDTGFVPKEYSFANYITNAFGQFDNYTPMQLAQYVATIANDGVRVAPRIVEGIYGNN
DKGGDLIQQLQPTEMNKVNISDSDMSILHQGFYQVAHGTSGLTTGRAFSNGALVSISGK
TGESYVAGSQEANNTNAVAYAPSDNPQIAVAVVFPHNTN

>JP_2b12

TNVFVPGSVVKAATISSGWENGVLSGNQTLTDQPIVFQGSAPIYSWYKLAYGSFP
ITAVLEYSSNAYMVQTALGIMGQTYQPNMFVGTSNLETAMGKLRATFGEYGLGVATGI
DLPDTGFVPKEYSFANYITNAFGQFDNYTPMQLAQYVATIANDGVRVAPRIVEGIYGNN
DKGGELIQAIDTKEINKVNISESDMAILHQGFYQVSHGTSPLTTGRAFSDGATVSISGKTG
ESYVAGGQEANNTNAVAYAPTENPQIAVAVVFPHNTN

>JP_2b13

TNVFVPGSVVKAATISSGWENGVLSGNQTLTDRSIVFQGSAPINSWYTQAYGSFP
ITAVLEYSSNAYMVQTALGLMGQTYQPNMFVGTSNLESAMGKLRSTFGEYGLGSATGI
DLPDTGFIPKDYSFANYITNAFGQFDNYTPMQLAQYVATIANDGVRVAPRIVEGIYGNN
DKGGDLIQQLQPTEMNKVNISDSDMSVLHQGFYQVAHGTSGLTTGRAFSNGASVSISGK
TGESYVEGGQEANNTNAVAYAPSDNPQIAVAVVFPHNTN

>JP_2b14

TNVFVPGSVVKAATISSGWENGVLSGNQTLTDQSIVFQGSAPINSWYTQAYGSFP
ITAVLEYSSNAYMVQTALGIMGQTYQPNMFVLTNNLESAMGKLRSTFAEYGLGASTGI
DLPDTGFIPKEYNFANYITNAFGQFDNYTPMQLAQYVATIANDGVRVAPRIVEGIYGNN
DKGGDLIQQLQPTEMNKVNISDSDMSVLHQGFYQVAHGTSGLTTGRAFSNGALVSISGK
TGESYVADGQQATNTNAVAYAPSDNPQIAVAVVFPHNTN

>JP_2b15

TNVFVPGSVVKAATISSGWENGVLSGNQTLTDQPIVFQDSAPIYSWYKLAYGSFP
ITAVLEYSSNAYMVQTALGIMGQTYQPNMFVGTSNLESAMEKLRSTFGEYGLGTATGID
LPDTGFVPKEYSFANYITNAFGQFDNYTPMQLAQYVATIANNGVRVAPRIVEGIYGNND
KGGDLIQQLQPTEMNKVNISDSDMSILHQGFYQVAHGTSGLTTGRAFSNGAAVSISGKT
GESYVEGGQEANNTNAVAYAPSDNPQIAVAVVFPHNTN

>JP_2b16

TNVFVPGSVVKAATISSGWENGVLSGNQTLTDQPIVFQGSAPIYSWYKLAYGSFP
ITAVLEYSSNAYMVQTALGIMGQTYQPNMFVGTSNLETAMGKLRATFGEYGLGAATGI
DLPDTGFVPKEYSFANYITNAFGQFDNYTPMQLAQYVATIANNGVRVAPRIVEGIYGNN
DKGGDLIQQLQPTEMNKVNISDSDMSILHQGFYQVAHGTSGLTTGRAFSNGASVSISGK
TGESYVEGGQEANNTNAVAYAPSDNPQIAVAVVFPHNTN

>JP_2b17

TNVFVPGSVVKAATISSGWENGVLSGNQTLTDQSIVFQGSAPINSWYTQAYGSFP
ITAVLEYSSNTYMVQTALGLMGQTYQPNMFVGTSNLESAMEKLRSTFGEYGLGTATGI
DLPDTGFVPKEYSFANYITNAFGQFDNYTPMQLAQYVATIANDGVRVAPRIVEGIYGNN
DKGGDLIQQLQPTEINKVNISDSDMSILHQGFYQVAHGTSGLTTGRAFSNGALVSISGKT
GESYVADGQQATNTNAVAYAPSDNPQIAVAVVFPHNTN

>JP_2b18

TNVFVPGSVVKAATISSGWENGVLSGNQTLTDQSIFFQGSAPINSWYTQAYGSFPI
TAVLEYSSNTYMVQTALGLMGQTYQPNMFVGTSNLESAMEKLRSTFGEYGLGTATGID
LPDTGFVPKEYSFANYITNAFGQFDNYTPMQLAQYVATIANNGVRVAPRIVEGIYGNND

KGGDLIQQLQPTEMNKVNISDSDMSILHQGFYQVAHGTSGLTTGRAFSNGALVSISGKTGESYVADGQQATNTNAVAYAPSDNPQIAVAVVFPHNTN

>JP_2b19

TNVFVPGSVVKAATISSGWENGVLSGNQTLTDQSIVFQGSAPINSWYTQAYGSFPITAVLEYSSNTYMVQTALGLMGQTYQPNMFVGTSNLESAMEKLRSTFGEYGLGTATGIDLPDTGFVPKEYSFANYITNAFGQFDNYTPMQLAQYVATIANNGVRVAPRIVEGIYGNNDKGGDLIQQLQPTEMNKVNISDSDMSILHQGFYQVAHGTSALTTGRAFSNGAAVSISGKTGESYVAGGQKADNTNAVAYAPSDNPQIAVAVVFPHNTN

>JP_2b20

TNVFVPGSVVKAATISSGWENGVLSGNQTLTDQSIVFQGSAPINSWYTQAYGSFPITAVLEYSSNTYMVQTALGLMGQTYQPNMFVGTSNLESAMEKLRSTFGEYGLGTATGIDLPDTGFVPKEYSFANYITNAFGQFDNYTPMQLAQYVATIANNGVRVAPRIVEGIYGNNDKGGDLIQQLQPTEMNKVNISDSDMSILHQGFYQVAHGTSELTTGRAFLNGALVSISGKTGESYVADGQQATNTNAVAYAPSDNPQIAVAVVFPHNTN

>JP_2b21

TNVFVPGSVVKAATISSGWENGVLSGNQTLTDQSIVFQGSAPINSWYTQAYGSFPITAVLEYSSNSYMVQTALGLMGQTYQPNMFVGTSNLESAMEKLRSTFGEYGLGTATGIDLPDTGFVPKEYSFANYITNAFGQFDNYTPMQLAQYVATIANNGVRVAPRIVEGIYGNNDKGGDLIQQLQPTEMNKVNISDSDMSILHQGFYQVAHGTSGLTTGRAFSNGALVSISGKTGESYVADGQQATNTNAVAYAPSDNPQIAVAVVFPHNTN

pbp2x

>JP_2x1

GTDGIITYEKDRLGNIVPGTEQVSQQTVDGKDVYTTISSPLQSFMETQMDAFQEKVKGKTATLVSAKTGEILATTQRPTFDADTKEGITEDFVWRDILYQSNYEPGSPMKVMMLAAANNTFPGGEVFNSSELKIADATIRDWDVNEGLTGGRMMTFSQGFAHSSNVGMTLLEQKMATWLDYLNRFKFGVPTRFGLTDEYAGQLPADNIVNIAMSAFGQGISVTQTQMLRAFTANDGVMLEPKFISALYDPNDQSVRKSQKEIVGNPVSKEAASVTRDHMVMVGTDPTYGTMHSTGKATVNVPGQNVALKSGTAEIADEKNGGYLTGSTNNIFSVVSMHPAENPDFILYV

>JP_2x2

GKDGIITYEKDRLGNIVPGTEQVSQQTVDGKDVYTTISSTLQSFMETQMDAFLEK VKGKTATLVSAKTGEILATTQRPTFNADTKEGITEDFVWRDILYQSNYEPGSAMKVMTL ASSNNTFPSGEYFNSSEFKIADATTRDWDVNDGLTTGGMMTFLQGFAHSSNVGMSLLE QKMATWLDYLSRFKFGVPTRFGLTDEYAGQLPADNIVSIAQSSFGQGISVTQTQMLRAF TANDGVMLEPKFISAIYDTNNQSVRKSQKEIVGNPVSKEAASTTRNHMILVGTDPLYGT MHYTGKPIITVPGQNVAVKSGTAQIADEKNGGYLVGSTNYIFSVVTMNPAENPDFILYV

>JP_2x3

GKDGIITYEKDRLGNIVPGTEQVSQQTVDGKDVYTTLSSPLQSFMETQMDAFLEK VKGKTATLVSAKTGEILATTQRPTFNADTKEGITEDFVWRDILYQSNYEPGSAFKVMML ASSNNTFPSGEYFNSSEFKIADATTRDWDVNEGLTTGGMMTFSQGFAHSSNVGTSLLEQ KMATWLDYLKRFKFGVPTRFGLTDEYAGQLPADNIVSIAQSSFGQGISVTQTQMLRAFT ANDGVMLEPKFISAIYDTNNQSVRKSQKEIVGNPVSKEAASTTRNHMILVGTDPLYGTM HYTGKPIITVPGQNVAVKSGTAQIADEKNGGYLVGSTNYIFSVVTMNPAENPDFILYV

>JP_2x4

GTDGIITYEKDRVGNIVPGTELVSQQTVDGKDVYTTLSSPLQSFMETQMDAFLEK VKGKTATLVSAKTGEILATTQRPTFNADTKEGITEDFVWRDILYQSNYEPGSAMKVMTL AASNNTFPSGEYFNSSEFKIADATTRDWDVNEGLTTGGMMTFLQGFAHSSNVGMSLLE QKMATWLDYLKRFKFGVPTRFGLTDEYAGQLPADNIVSIAQSSFGQGISVTQTQMLRAF TANDGVMLEPKFISAIYDTNNQSVRKSQKEIVGNPVSKEAASTTRNHMILVGTDPLYGT MHYTGKPIITVPGQNVAVKSGTAQIADEKNGGYLVGSTNYIFSAVTMNPAENPDFILYV

>JP_2x5

GTDGIITYEKDRLGNIVPGTELVSQQTVDGKDVYTTLSSPLQSFMETQMDAFLEK VKGKTATLVSAKTGEILATTQRPTFNADTKEGITEDFVWRDILYQSNYEPGSAMKVMTL ASSNNTFPSGEYFNSSEFKIADATTRDWDVNEGLTTGGMMTFLQGFAHSSNVGMSLLE QKMATWLDYLKRFKFGVPTRFGLTDEYAGQLPADNIVSIAQSSFGQGISVTQTQMLRAF TANDGVMLEPKFISAIYDTNNQSVRKSQKEIVGNPVSKEAASTTRNHMILVGTDPLYGT MHYTGKPIITVPGQNVAVKSGTAQIADEKNGGYLVGSTNYIFSAVTMNPAENPDFILYV

>JP_2x6

GKDGIITYEKDRLGNIVPGTEQVSQQTVDGKDVYTTLSSPLQSFMETQMDAFLEK
VKGKTATLVSAKTGEILATTQRPTFNADTKEGITEDFVWRDILYQSNYEPGSAFKVMML
ASSNNTFPSGEYFNSSEFKIADATTRDWDVNAGLTTGGMMTFLQGFVHSSNVATSLLEQ
KMATWLDYLKRFKFGVPTRFGLTDEYAGQLPADNIVSIAQSSFGQGISVTQTQMLRAFT
ANDGVMLEPKFISAIYDTNNQSVRKSQKEIVGNPVSKEAASTTRNHMILVGTDPLYGTM
HYTGKPIITVPGQNVAVKSGTAQIADEKNGGYLVGSTNYIFSVVTMNPAENPDFILYV

>JP_2x7

GTDGIITYEKDRLGNIVPGTEQVSQQTVDGKDVYTTLSSPLQSFMETQMDAFLEK
VKGKTATLVSAKTGEILATTQRPTFNADTKEGITEDFVWRDILYQSNYEPGSAMKVMTL
ASSNNTFPSGEYFNSSEFKIADATTRDWDVNAGLTTGGMMTFLQGFAHSSNVGMSLLE
QKMATWLDYLKRFKFGVPTRFGLTDEYAGQLPADNIVSIAQSSFGQGISVTQTQMLRAF
TANDGVMLEPKFISAIYDTNNQSVRKSQKEIVGNPVSKEAASTTRNHMILVGTDPIYGT
MHYTGKPIITVPGQNVAVKSGTAQIADEKNGGYLVGSTNYIFSVVTMNPAENPDFILYV

>JP_2x8

GKDGIITYEKDRLGNIVPGTEQVSQQTVDGKDVYTTISSTLQSFMETQMNAFQEK
VKGKTATLVSAKTGEILATTQRPTFDADTKEGLTKDFVWRDILYQSNYEPGSAMKVMT
LAAANNTFPGGEVFNSSELKVADVTTRDWDVNEGLTGGGMMTFSQGFAHSSNVGMTL
LEQKMATWLDYLNRFKFGVPTRFGLTDEYAGQLPADNIVNIAQSSFGQGISVTQTQMIR
AFTANDGVMLEPKFISAIYDPNDQTARKSQKEIVGNPVSKDAASLTRTHMVLVGTDPVY
GTMHKTGKPTVTVPGQNVALKSGTAQIADEKNGGYLVGLTNYIFSAVSMNPAENPDFIL
YV

>JP_2x9

GKDGIITYEKDRLGNIVPGTEQVSQQTVDGKDVYTTISSTLQSFMETQMNVFQEK
VKGKTATLVSAKTGEILATTQRPTFDADTKEGLTKDFVWRDILYQSNYEPGSTMKVMT
LAAANNTFPSGEYFNSSELKIADVTIRDWDVNDGLTTGRMMTFLQGFALSSNVGMTLL
EQKMATWLDYLNRFKFGVPTRFGLTDEYAGQLPADNIVNIAQSSFGQGISVTQTQMIRA
FTANDGVMLEPKFISAIYDPNDQTARKSQKEIVGNPVSKDAASLTRTNMVLVGTDPVYG

TMHSTGKPTVTVPGQNVALKSGTAQIADEKNGGYLVGLTNYIFSAVSMSPAENPDFILY
V

>JP_2x10

GTDGIITYEKDRLGNIVPGTEQVSQQTVDGKDVYTTISSTLQSFMETQMNAFQEK
VKGKTATLVSAKTGEILATTQRPTFDADTKEGLTKDFVWRDILYQSNYEPGSTMKVMT
LAAANNTFPGGEVFNSSELKIADVTIRDWDVNDGLTTGRMMTFLQGFALSSNVGMSLL
EQKMTTWLDYLNRFKFGVPTRFGLTDEYAGQLPADNIVNIAQSSFGQGISVTQTQMLRA
FTANDGVMLEPKFISAIYDPNDQTARKSQKEIVGNPVSKDAASLTRTNMVLVGTDPVYG
TMHSTGKPTVTVPGQNVALKSGTAQIADEKNGGYLVGLTNYIFSAVSMNPAENPDFILY
V

>JP_2x11

GTDGIITYEKDRLGNIVPGTEQVSQQTVDGKDVYTTISSPLQSFMETQMDAFQEK
VKGKTATLVSAKTGEILATTQRPTFDADTKEGITEDFVWRDILYQSNYEPGSTMKVMML
AAANNTFPGGEVFNSSELKIADATIRDWDVNEGLTGGRMMTFSQGFAHSSNVGMTLLE
QKMATWLDYLNRFKFGVPTRFGLTDEYAGQLPADNIVNIAMSAFGQGISVTQTQMLRA
FTANDGVMLEPKFISALYDPNDQSVRKSQKEIVGNPVSKEAASVTRDHMVMVGTDPTY
GTMHSTGKATVNVPGQNVALKSGAAEIADEKNGGYLTGSTNNIFSVVSMHPAENPDFIL
YV

>JP_2x12

GTDGIITYEKDRLGNIVPGTEQVSQQTVDGKDVYTTISSPLQSFMETQMDAFQEK
VKGKTATLVSAKTGEILATTQRPTFDADTKEGITEDFVWRDILYQSNYEPGSTMKVMML
AAANNTFPGGEVFNSSELKIADATIRDWDVNEGLTGGRMMTFSQGFAHSSNVGMTLLE
QKMATWLDYLNRFKFGVPTRFGLTDEYAGQLPADNIVNIAMSAFGQGISVTQTQMLRA
FTANDGVMLEPKFISALYDPNDQSVRKSQKEIVGNPVSKEAASVTRDHMVMVGTDPTY
GSMHSTGKATVNVPGQNVALKSGTAEIADEKNGGYLTGSTNNIFSVVSMHPAENPDFIL
YV

>JP_2x13

GTDGIITYEKDRLGNIVPGTEQVSQQTVDGKDVYTTISSPLQSFMETQMDAFQEK
VKGKTATLVSAKTGEILATTQRPTFDADTKEGITEDFVWRDILYQSNYEPGSTMKVMML

AAANNTFPGGEVFNSSELKIADATIRDWDVNEGLTGGRMMTFSQGFAHSSNVGMTFLE
QKMATWLDYLNRFKFGVPTRFGLTDEYAGQLPADNIVNIAMSAFGQGISVTQTQMLRA
FTANDGVMLEPKFISALYDPNDQSVRKSQKEIVGNPVSKEAASVTRDHMVMVGTDPTY
GTMHSTGKATVNVPGQNVALKSGTAEIADEKNGGYLTGSTNNIFSVVSMHPAENPDFIL
YV

>JP_2x14

GTDGIITYEKDRLGNIVPGTEQVSQQTVDGKDVYTTISSTLQSFMETQMNAFQEK
VKGKTATLVSAKTGEILATTQRPTFDADTKEGLTKDFVWRDILYQSNYEPGSTMKVMT
LAASNNTFPGGEYFNSSELKIADVTIRDWDVNDGLTTGRMMTFLQGFALSSNVGMSLLE
QKMTTWLDYLNRFKFGVPTRFGLTDEYAGQLPADNIVNIAQSSFGQGISVTQTQMIRAF
TANDGVMLEPKFISAIYDTNNQSVRKSQKEIVGKPVSEDAASLTRTNMILVGTDPLYGT
MHQTGKPIITVPGQNVAVKSGTAQIADEKNGGYLVGSTNYIFSVVTMNPAENPDFILYV

>JP_2x15

GTDGIITYEKDRLGNIVPGTEQVSQRTMDGKDVYTTISSPLQSFMETQMDAFQEK
VKGKTATLVSAKTGEILATTQRPTFDADTKEGLTKDFVWRDILYQSNYEPGSTMKVMT
LASSNNTFPSGEYFNSSELKIADVTIRDWDVNDGLTTGRMMTFLQGFALSSNVGMSLLE
QKMTTWLDYLNRFKFGVPTRFGLTDEYAGQLPADNIVNIAQSSFGQGISVTQTQMLRAF
TANDGVMLEPKFISAIYDTNNQSVRKSQKEIVGKPVSEDAASLTRTNMILVGTDPIYGTM
HYTGKPIITVPGQNVAVKSGTAQIADEKNGGYLVGSTNYIFSVVTMNPAENPDFILYV

>JP_2x16

GTDGIITYEKDRVGNIVPGTELVSQQTVDGKDVYTTLSSPLQSFMETQMDAFLEK
VKGKTATLVSAKTGEILATTQRPTFNADTKEGITEDFVWRDILYQSNYEPGSAMKVITLA
SSNNTFPSGEYFNSSEFKIADATTRDWDVNEGLTTGGMMTFLQGFAHSSNVGMSLLEQ
KMATWLDYLKRFKFGVPTRFGLTDEYAGQLPADNIVSIAQSSFGQGISVTQTQMLRAFT
ANDGVMLEPKFISAIYDTNNQSVRKSQKEIVGKPVSEDTASLTRTNMILVGTDPLYGTM
HYTGKPIITVPGQNVAVKSGTAQIADEKNGGYLVGSTNYIFSAVTMNPAENPDFILYV

>JP_2x17

GTDGIITYEKDRLGNIVPGTELVSQQTVDGKDVYTTLSSPLQSFMETQMDAFLEK
VKGKTATLVSAKTGEILATTQRPTFNADTKEGITKDFVWRDILYQSNYEPGSAMKVMTL

AAANNTFPSGEYFNSSEFKIADATTRDWDVNAGLTTGGMMTFLQGFAHSSNVGMSLLE
QKMATWLDYLKRFKFGVPTRFGLTDEYAGQLPADNIVSIAQSSFGQGISVTQTQMLRAF
TANDGVMLEPKFISAIYDTNNQSVRKSQKEIVGNPVPKEAASTTRNHMILVGTDPLYGT
MHYTGKPIITVPGQNVAVKSGTAQIADEKNGGYLVGSTNYIFSAVTMNPAENPDFILYV

>JP_2x18

GTDGIITYEKDRLGNIVPGTEQVSQQTVDGKDVYTTLSSPLQSFMETQMDAFLEK
VKGKTATLVSAKTGEILATTQRPTFNADTKEGITEDFVWRDILYQSNYEPGSAFKVMML
ASSNNTFPSGEYFNSSEFKIADATTRDWDVNAGLTTGGMMTFLQGFAHSSNVGTSLLEQ
KMATWLDYLKRFKFGVPTRFGLTDEYAGQLPADNIVSIAQSSFGQGISVTQTQMLRAFT
ANDGVMLEPKFISAIYDTNNQSVRKSQKEIVGKPVSEDAASTTRNHMILVGTDPLYGTM
HYTGKPIITVPGQNVAVKSGTAQIADEKNGGYLVGSTNYIFSVVTMNPAENPDFILYV

>JP_2x19

GTDGIITYEKDRLGNIVPGTEQVSQQTVDGKDVYTTLSSPLQSFMETQMDAFLEK
VKGKTATLVSAKTGEILATTQRPTFNADTKEGITEDFVWRDILYQSNYEPGSAFKVMML
ASSNNTFPSGEYFNSSEFKMADVTTRDWDVNGGLTTGGMMTFLQGFAHSSNVGMSLLE
QKMATWLDYLKRFKFGVPTRFGLTDEYAGQLPADNIVNIAQSSFGQGISVTQTQMLRAF
TANDGVMLEPKFISAIYDTNNQSVRKSQKEIVGKPVSEDAASTTRNHMILVGTDPLYGT
MHYTGKPIITVPGQNVAVKSGTAQIADEKNGGYLVGSTNYIFSVVTMNPAENPDFILYV

>JP_2x20

GTDGIITYEKDRLGNIVPGTEQISQQTVDGKDVYTTISSPLQSFMETQMDAFQEKV
KGKTATLVSAKTGEILATTQRPTFNADTKEGITKDFVWRDILYQSNYEPGSAFKVMMLA
SSNNTFPSGEYFNSSEFKIADATTRDWDVNAGLTTGGMMTFLQGFAHSSNVGTSLLEQK
MATWLDYLKRFKFGVPTRFGLTDEYAGQLPADNIVSIAQSSFGQGISVTQTQMLRAFTA
NDGVMLEPKFISAIYDTNNQSVRKSQKEIVGNPVSKEAASTTRNHMILVGTDPLYGTMH
YTGKPIITVPGQNVAVKSGTAQIADEKNGGYLVGSTNYIFSVVTMNPAENPDFILYV

>JP_2x21

GTDGIITYEKDRLGNIVPGTELVSQQTVDGKDVYTTLSSPLQSFMETQMDAFLEK
EKGKTATLVSAKTGEILATTQRPTFNADTKEGITEDFVWRDILYQSNYEPGSGMKAMTL
ASSNNTFPSGEYFNSSEFKIADVTTRDWDVNEGLTTGGMMTFLQGFAHSSNVGMSLLE

QKMATWLDYLKRFKFGVPTRFGLTDEYAGQLPADNIVSIAQSSFGQGISVTQTQMLRAF TANDGVMLEPKFISAIYDTNNQSVRKSQKEIVGNPVSKEAASTTRNHMILVGTDPLYGT MHYTGKPIITVPGQNVAVKSGTAQIADEKNGGYLVGSTNYIFSVVTMNPAENPDFILYV

>JP_2x22

GKDGIITYEKDRLGNIVPGTEQVSQQTVDGKDVYTTLSSPLQSFMETQMDAFLEK VKGKTATLVSAKTGEILATTQRPTFNADTKEGITEDFVWRDILYQSNYEPGSAMKVMTL ASSNNTFPSGEYFNSSEFKIADATTRDWDVNDGLTTGGMMTFLQGFAHSSNVGMSLLE QKMATWLDYLKRFKFGVPTRFGLTDEYAGQLPADNIVSIAQSSFGQGISVTQTQMLRAF TANDGVMLEPKFISAIYDTNNQSVRKSQKEIVGNPVSKEAASTTRNHMILVGTDPLYGT MHYTGKTIITVPGQNVAVKSGTAQIADEKNGGYLVGSTNYIFSVVTMNPAENPDFILYV

>JP_2x23

GTDGIITYEKDRLGNIVPGTEQVSQRTMDGKDVYTTISSPLQSFMETQMDAFLEK VKGKTATLVSAKTGEILATTQRPTFNADTKEGITKDFVWRDILYQSNYEPGSTMKVMTL AAANNTFPGGEVFDSSELKIADVTTRDWDVNEGLTGGRMMTFSQGFALSSNVGMTLLE QKMATWLDYLNRFKFGVPTRFGLTDEYAGQLPADNIVNIAQSSFGQGISVTQTQMIRAF TANDGVMLEPKFISAIYDPNDQTARKSQKEIVGNPVSKDAASLTRTHMVLVGTDPVYGT MHSTGKPTVTVPGQNIALKSGTAQIADEKNGGYLVGTTNHIFSAVSMNPAENPDFILYV

>JP_2x24

GTDGIITYEKDRLGNIVPGTEQVSQRTMDGKDVYTTISSPLQSFMETQMDAFQEK VKGKTATLVSAKTGEILATTQRPTFDADTKEGITKDFVWRDILYQSNYEPGSTMKVMTL AAANNTFPGGEVFNSSELKIADVTIRDWDVNDGLTTGRMMTFLQGFALSSNVGMSLLE QKMATWLDYLNRFKFGVPTRFGLTDEYAGQLPADNIVNIAQSSFGQGISVTQTQMLRAF TANDGVMLEPKFISAIYDPNDQTARKSQKEIVGNPVSKDAASLTRTNMVLVGTDPVYGT MHQTGKPIITVPGQNVAVKSGTAQIADEKNGGYLVGSTNYIFSVVTMNPAENPDFILYV

>JP_2x25

GTDGIITYEKDRLGNIVPGTEQVSQQTVDGKDVYTTISSPLQSFMETQMDAFQEK VKGKTATLVSAKTGEILATTQRPTFDADTKEGLTKDFVWRDILYQSNYEPGSAMKVMT LASSNNTFPSGEYFNSSEFKIADATTRDWDVNDGLTTGGMMTFLQGFAHSSNVGMSLL EQKMATWLDYLKRFKFGVPTRFGLTDEYAGQLPADNIVSIAQSSFGQGISVTQTQMLRA

FTANDGVMLEPKFISAIYDTNNQSVRKSQKEIVGNPVSKEAASTTRNHMILVGTDPLYG
TMHYTGKPIITVPGQNVAVKSGTAQIADEKNGGYLVGSTNYIFSAVTMNPAENPDFILY
V

>JP_2x26

GKDGIITYEKDRLGNIVPGTEQVSQQTVDGKDVYTTISSTLQSFMETQMDAFLEK
LKGKTATLVSAKTGEILATTQRPTFNADTKEGITEDFVWRDILYQSNYEPGSAMKVMTL
ASSNNTFPSGEYFNSSEFKIADATTRDWDVNDGLTTGGMMTFLQGFAHSSNVGMSLLE
QKMATWLDYLSRFKFGVPTRFGLTDEYAGQLPADNIVSIAQSSFGQGISVTQTQMLRAF
TANDGVMLEPKFISAIYDTNNQSVRKSQKEIVGNPVSKEAASTTRNHMILVGTDPLYGT
MHYTGKPIITVPGQNVAVKSGTAQIADEKNGGYLVGSTNYIFSVVTMNPAENPDFILYV

>JP_2x27

GTDGIITYEKDRLGNIVPGTEQVSQRTMDGKDVYTTISSPLQSFMETQMDAFQEK
VKGKTATLVSAKTGEILATTQRPTFDADTKEGITEDFVWRDILYQSNYEPGSTMKVMML
AAANNTFPGGEVFNSSELKIADATIRDWDVNEGLTGGRMMTFSQGFAHSSNVGMTLLE
QKMATWLDYLNRFKFGVPTRFGLTDEYAGQLPADNIVNIAQSSFGQGISVTQTQMLRAF
TANDGVMLEPKFISALYDPNDQSVRKSQKEIVGNPVSKAAASSTREHMVMVGTDPVYG
TMHSTGKPNVNVPGQNVALKSGTAEIADEKNGGYLTGETNNIFSVVSMHPAENPDFILY
V

>JP_2x28

GTDGIITYEKDRLGNIVPGTEQASQHTVDGKDVYTTLSSPLQSFMETQMDAFQEK
LKGKTATLVSAKTGEILATTQRPTFNADTKDGITKDFVWRDILYQSNYEPGSAMKVMTL
ASANNTFPGGEYFNSSELKIADATIRDWDVNDGLTTGGMMTFSQGFAHSSNVGMSLLE
QKMATWLDYLNRFKFGVPTRFGLTDEYTGQLPADNIVNIAMSAFGQGISVTQTQMLRA
FTANDGVMLEPKFISALYDPNDQSVRKSQKEIVGNPVSKAAASSTREHMVMVGTDPVY
GTMHSTGKPNVNVPGQNVALKSGTAQIADEKNGGYLTGETNYIFSVVSMHPAENPDFIL
YV

>JP_2x29

GTDGIITYEKDRLGNIVPGTEQASQHTVDGKDVYTTLSSPLQSFMETQMDAFQEK
EKGKTATLVSAKTGEILATTQRPTFNADTKDGITKDFVWRDILYQSNYEPGSAMKVMTL

ASANNTFPGGEYFNSSELKIADATIRDWDVNDGLTTGGMMTFSQGFAHSSNVGMSLLE
QKMATWLDYLNRFKFGVPTRFGLTDEYTGQLPADNIVNIAMSAFGQGISVTQTQMLRA
FTANDGVMLEPKFISALYDPNDQSVRKSQKEIVGNPVSKAAASSTREHMVMVGTDPVY
GTMHSTGKPNVNVPGQNVALKSGTAQIADEKNGGYLTGETNYIFSVVSMHPAENPDFIL
YV

>JP_2x30

GTDGIITYEKNRLGNIVPGTEQASQHTVDGKDVYTTLSSPLQSFMETQMDAFQEK
VKGKTATLVSAKTGEILATTQRPTFNADTKDGITKDFVWRDILYQSNYEPGSAMKVMT
LASANNTFPGGEYFNSSELKIADATIRDWDVNDGLTTGGMMTFSQGFAHSSNVGMSLL
EQKMATWLDYLNRFKFGVPTRFGLTDEYTGQLPADNIVNIAMSAFGQGISVTQTQMLR
AFTANDGVMLEPKFISALYDPNDQSVRKSQKEIVGNPVSKAAASSTREHMVMVGTDPV
YGTMHSTGKPNVNVPGQNVALKSGTAQIADEKNGGYLTGETNYIFSVVSMHPAENPDF
ILYV

>JP_2x31

GTDGIITYEKDRLGNIVPGTEQASQHTVDGKDVYTTLSSPLQSFMETQMDAFQEK
VKGKTATLVSAKTGEILATTQRPTFNADTKDGITKDFVWRDILYQSNYEPGSAMKVMT
LASANNTFPGGEYFNSSELKIADATIRDWDVNDGLTTGGMMTFSQGFAHSSNVGMSLL
EQKMATWLDYLNRFKFGVPTRFGLTDEYTGQLPADNIVNIAMSAFGQGISVTQTQMLR
AFTANDGVMLEPKFISALYDPNDQSVRKSQKEIVGNPVSKAAASSTREHMVMVGTDPV
YGTMHSTGKPNVNVPGQNVALKSGTAQIADEKNGGYLTGETNYIFSVVSMHPAENPDF
ILYV

>JP_2x32

GTDGIITYEKDRLGNIVPGTEQASQHTVDGKDVYTTLSSPLQSFMETQMDAFQEK
VKGKTATLVSAKTGEILATTQRPTFNADTKDGITKDFVWRDILYQSNYEPGSAMKVMT
LASANNTFPGGEYFNSSELKIADATIRDWDVNDGLTTGGMMTFSQGFAHSSNVGMSLL
EQKMATWLDYLNRFKFGVPTRFGLTDEYTGQLPADNIVNIAMSAFGQGISVTHTQMLR
AFTANDGVMLEPKFISALYDPNDQSVRKSQKEIVGNPVSKAAASSTREHMVMVGTDPV
YGTMHSTGKPNVNVPGQNVALKSGTAQIADEKNGGYLTGETNYIFSVVSMHPAENPDF
ILYV

>JP_2x33

GTDGIITYEKDRLGNIVPGTEQVSQQTVDGKDVYTTISSPLQSFMETQMDAFQEK
VKGKTATLVSAKTGEILATTQRPTFDADTKEGLTKDFVWRDILYQSNYEPGSTMKVMT
LAAANNTFPGGEVFDSSELKIADVTIRDWDVNEGLTGGRMMTLSQGFALSSNVGMTLL
EQKMATWLDYLNRFKFGVPTRFGLTDEYAGQLPADNIVNIAQSSFGQGISVTQTQMLR
AFTANDGVMLEPKFISALYDPNDQSVRKSQKEIVGNPVSKDAASLTRTNMVLVGTDPV
YGTMHSTGKPTVTVPGQNVALKSGTAQIADEKNGGYLVGLTDYIFSAVSMSPAENPDFI
LYV

>JP_2x34

GKDGIITYEKDRLGNIVPGTEQVFQQTVDGKDVYTTISSTLQSFMETQMNAFQEK
VKGKTATLVSAKTGEILATTQRPTFDADTKEGLTKDFVWRDILYQSNYEPGSTMKVMT
LAAANNTFPGGEVFDSSELKIADVTIRDWDVNEGLTGGRMMTFSQGFVLSSNVGMTLL
EQKMATWLDYLNRFKFGVPTRFGLTDEYAGQLPADNIVNIAQSSFGQGISVTQTQMIRA
FTANDGVMLEPKFISAIYDPNDQTARKSQKEIVGNPVSKDAASLTRTNMVLVGTDPVYG
TMHSTGKPTVTVPGQNVALKSGTAQIADEKNGGYLVGLTDYIFSAVSMSPAENPDFILY
V

>JP_2x35

GTDGIITYEKDRLGNIVPGTEQVSQQTVNGKDVYTTLSSPLQSFMETQMNAFQEK
VKGKTATLVSAKTGEILATTQRPTFDADTKEGLTKDFVWRDILYQSNYEPGSTMKVMT
LAAANNTFPGGEVFDSSELKIADVTIRDWDVNEGLTGGRMMTFSQGFALSSNVGMTLL
EQKMATWLDYLNRFKFGVPTRFGLTDEYAGQLPADNIVNIAQSSFGQGISVTQTQMIRA
FTANDGVMLEPKFISAIYDPNDQTARKSQKEIVGNPVSKDAASLTRTHMVLVGTDPVYG
TMHSTGKPTVTVPGQNVALKSGTAQIADEKNGGYLVGLTNYIFSAVSMSPAENPDFILY
V

>JP_2x36

GTDGIITYEKDRLGNIVPGTEQVSQQTVNGKDVYTTLSSPLQSFMETQMDAFLEK
VKGKTATLVSAKTGEILATTQRPTFNADTKEGITKDFVWRDILYQSNYEPGSTMKVMTL
AAANNTFPGGEVFDSSELKIADVTTRDWDVNEGLTGGRMMTFSQGFALSSNVGMTLLE
QKMATWLDYLNRFKFGVPTRFGLTDEYAGQLPADNIVNIAQSSFGQGISVTQTQMIRAF

TANDGVMLEPKFISAIYDPNDQTARKSQKEIVGNPVSKDAASLTRTHMVLVGTDPVYGT
MHSTGKPTVTVPGQNIALKSGTAQIADEKNGGYLVGTTNHIFSAVSMNPAENPDFILYV

>JP_2x37

GKDGIITYEKDRLGNIVPGTEQVSQQTVDGKDVYTTISSTLQSFMETQMNAFQEK
VKGKTATLVSAKTGEILATTQRPTFDADTKEGLTKDFVWRDILYQSNYEPGSTMKVMT
LAAANNTFPGGEVFDSSELKIADVTIRDWDVNEGLTGGRMMTFSQGFVLSSNVGMTLL
EQKMATWLDYLNRFKFGVPTRFGLTDEYAGQLPADNIVNIAQSSFGQGISVTQTQMIRA
FTANDGVMLEPKFITALYDPNNQSVRKSQKEIVGNPVSKDAASLTRTHMLLVGTDPVY
GTMHSTGKPTVTVPGQNVALKSGTAQIADEKNGGYLIGSTNYIFSAVSMNPAENPDFIL
YV

>JP_2x38

GTDGIITYEKDRLGNIVPGTEQVSQQTVDGKDVYTTISSTLQSFMETQMDAFQEK
VKGKTATLVSAKTGEILATTQRPTFDADTKEGITEDFVWRDILYQSNYEPGSTMKVMTL
AAANNTFPGGEVFNSSELKIADATIRDWDVNEGLTGGRMMTFSQGFAHSSNVGMTLLE
QKMATWLDYLNRFKFGVPTRFGLTDEYAGQLPADNIVNIAQSSFGQGISVTQTQMIRAF
TANDGVMLEPKFISAIYDPNDQTARKSQKEIVGNPVSKDAASLTRTNMVLVGTDAVYG
TMHSTGKPTVTVPGQNVALKSGTAEIADEKNGGYLVGSTNNIFSVVAMNPAENPDFILY
V

>JP_2x39

GTDGIITYEKDRVGNIVPGTELVSQQTVDGKDVYTTLSSPLQSFMETQMDAFLEK
VKGKTATLVSAKTGEILATTQRPTFNADTKEGITEDFVWRDILYQSNYEPGSAMKVMTL
ASSNNTFPSGEYFNSSEFKIADATTRDWDVNAGLTTGGMMTFLQGFAHSSNVGMSLLE
QKMATWLDYLKRFKFGVPTRFGLTDEYAGQLPADNIVSIAQSSFGQGISVTQTQMLRAF
TANDGVMLEPKFISAIYDTNNQSVRKSQKEIVGNPVSKEAASTTRNHMILVGTDPLYGT
MHYTGKPIITVPGQNVAVKSGTAQIADEKNGGYLVGSTNYIFSAVTMNPAENPDFILYV

>JP_2x40

GTDGIITYEKDRVGNIVPGTELVSQQTVDGKDVYTTLSSPLQSFMETQMDAFLEK
VKGKTATLVSAKTGEILATTQRPTFNADTKEGITEDFVWRDILYQSNYEPGSAMKVMTL
ASSNNTFPSGEYFNSSEFKIADATTRDWDVNEGLTTGGMMTFLQGFAHSSNVGMSLLE

QKMATWLDYLKRFKFGVPTRFGLTDEYAGQLPADNIVSIAQSSFGQGISVTQTQMLRAF TANDGVMLEPKFISAIYDTNNQSVRKSQKEIVGNPVSKEVASTTRNHMILVGTDPIYGT MHYTGKPIITVPGQNVAVKSGTAQIADEKNGGYLVGSTNYIFSVVTMNPAENPDFILYV

>JP_2x41

GKDGIITYEKDRLGNIVPGTEQVSQQTVDGKDVYTTLSSPLQSFMETQMDAFLEK VKGKTATLVSAKTGEILATTQRPTFNADTKEGITEDFVWRDILYQSNYEPGSAMKVMTL ASSNNTFPSGEYFNSSEFKIADATTRDWDVNEGLTTGGMMTFLQGFAHSSNVGMSLLE QKMATWLDYLKRFKFGVPTRFGLTDEYAGQLPADNIVSIAQSSFGQGISVTQTQMLRAF TANDGVMLEPKFISAIYDTNNQSVRKSQKEIVGNPVSKEAASTTRNHMILVGTDPLYGT MHYTGKPIITVPGQNVAVKSGTAQIADEKNGGYLVGSTNYIFSAVTMNPAENPDFILYV

>JP_2x42

GTDGIITYEKDRLGNIVPGTEQVSQQTVDGKDVYTTLSSPLQSFMETQMDAFLEK VKGKTATLVSAKTGEILATTQRPTFNADTKEGITEDFVWRDILYQSNYEPGSAMKVMTL ASSNNTFPSGEYFNSSEFKIADATTRDWDVNDGLTTGGMMTFLQGFAHSSNVGMSLLE QKMATWLDYLKRFKFGVPTRFGLTDEYAGQLPADNIVSIAQSSFGQGISVTQTQMLRAF TANDGVMLEPKFISAIYDTNNQSVRKSQKEIVGNPVSKEAASTTRNHMILVGTDPLYGT MHYTGKPIITVPGQNVAVKSGTAQIADEKNGGYLVGSTNYIFSVVTMNPAENPDFILYV

>JP_2x43

GKDGIITYEKDRLGNIVPGTEQVSQQTVDGKDVYTTLSSPLQSFMETQMDAFLEK VKGKTATLVSAKTGEILATTQRPTFNADTKEGITEDFVWRDILYQSNYEPGSAMKVMTL ASSNNTFPSGEYFNSSEFKIADATTRDWDVNDGLTTGGMMTFLQGFAHSSNVGMSLLE QKMATWLDYLNRFKFGVPTRFGLTDEYAGQLPADNIVSIAQSSFGQGISVTQTQMLRAF TANDGVMLEPKFISAIYDTNNQSVRKSQKEIVGNPVSKEAASTTRNHMILVGTDPLYGT MHYTGKPIITVPGQNVAVKSGTAQIADEKNGGYLVGSTNYIFSVVTMNPAENPDFILYV

>JP_2x44

GKDGIITYEKDRVGNIVPGTELVSQQTVDGKDVYTTLSSPLQSFMETQMDAFLEK VKGKTATLVSAKTGEILATTQRPTFNADTKEGITEDFVWRDILYQSNYEPGSAFKVMML ASSNNTFPSGEYFNSSEFKMADVTTRDWDVNGGLTTGGMMTFLQGFAHSSNVGTSLLE QKMATWLDYLKRFKFGVPTRFGLTDEYAGQLPADNIVSIAQSSFGQGISVTQTQMLRAF

TANDGVMLEPKFISAIYDTNNQSVRKSQKEIVGNPVSKEAASTTRNHMILVGTDPLYGT
MHYTGKPIITVPGQNVAVKSGTAQIADEKNGGYLVGSTNYIFSVVTMNPAENPDFILYV

>JP_2x45

GKDGIITYEKDRLGNIVPGTEQVSQQTVDGKDVYTTLSSPLQSFMETQMDAFLEK
VKGKTATLVSAKTGEILATTQRPTFNADTKEGITEDFVWRDILYQSNYEPGSAFKVMML
ASSNNTFPSGEYFNSSEFKMADVTTRDWDVNGGLTTGGMMTFLQGFAHSSNVGTSLLE
QKMATWLDYLKRFKFGVPTRFGLTDEYAGQLPADNIVSIAQSSFGQGISVTQTQMLRAF
TANDGVMLEPKFISAIYDTNNQSVRKSQKEIVGNPVSKEAASTTRNHMILVGTDPLYGT
MHYTGKPIITVPGQNVAVKSGTAQIADEKNGGYLVGSTNYIFSVVTMNPAENPDFILYV

>JP_2x46

GKDGIITYEKDRLGNIVPGTEQVSQQTVDGKDVYTTLSSPLQSFMETQMDAFLEK
VKGKTATLVSAKTGEILATTQRPTFNADTKEGITEDFVWRDILYQSNYEPGSAFKVMML
ASSNNTFPSGEYFNSSEFKLADATTRDWDVNAGLTTGGMMTFLQGFVHSSNVATSLLE
QKMATWLDYLKRFKFGVPTRFGLTDEYAGQLPADNIVSIAQSSFGQGISVTQTQMLRAF
TANDGVMLEPKFISAIYDTNNQSVRKSQKEIVGNPVSKEAASTTRNHMILVGTDPLYGT
MHYTGKPIITVPGQNVAVKSGTAQIADEKNGGYLVGSTNYIFSVVTMNPAENPDFILYV

>JP_2x47

GKDGIITYEKDRLGNIVPGTEQVSQQTVDGKDVYTTLSSPLQSFMETQMDAFLEK
LKGKTATLVSAKTGEILATTQRPTFNADTKEGITEDFVWRDILYQSNYEPGSAFKVMML
ASSNNTFPSGEYFNSSEFKIADATTRDWDVNEGLTTGGMMTFLQGFAHSSNVGTSLLEQ
KMATWLDYLKRFKFGVPTRFGLTDEYAGQLPADNIVSIAQSSFGQGISVTQTQMLRAFT
ANDGVMLEPKFISAIYDTNNQSVRKSQKEIVGNPVSKEAASTTRNHMILVGTDPLYGTM
HYTGKPIITVPGQNVAVKSGTAQIADEKNGGYLVGSTNYIFSVVTMNPAENPDFILYV

>JP_2x48

GKDGIITYEKDRLGNIVPGTEQVSQQTVDGKDVYTTLSSPLQSFMETQMDAFLEK
VKGKTATLVSAKTGEILATTQRPTFNADTKEGITEDFVWRDILYQSNYEPGSAFKVMML
ASSNNTFPSGEYFNSSEFKLADATTRDWDVNGGLTTGGMMTFLQGFAHSSNVGMSLLE
QKMATWLDYLKRFKFGVPTRFGLTDEYAGQLPADNIVSIAQSSFGQGISVTQTQMLRAF

TANDGVMLEPKFISAIYDTNNQSVRKSQKEIVGKPVSEDAASTTRNHMILVGTDPLYGT MHYTGKPIITVPGQNVAVKSGTAQIADEKNGGYLVGSTNYIFSVVTMNPAENPDFILYV

>JP_2x49

GTDGIITYEKDRLGNIVPGTELVSQQTVDGKDVYTTLSSPLQSFMETQMDAFLEK VKGKTATLVSAKTGEILATTQRPTFNADTKEGITEDFVWRDILYQSNYEPGSAMKVMTL ASSNNTFPSGEYFNSSEFKIADATTRDWDVNEGLTTGGMMTFLQGFAHSSNVGMSLLE QKMATWLDYLNRFKFGVPTRFGLTDEYAGQLPADNIVSIAQSSFGQGISVTQTQMLRAF TANDGVMLEPKFISAIYDTNNQSVRKSQKEIVGNPVSKEAASTTRNHMILVGTDPLYGT MHYTGKPIITVPGQNVAVKSGTAQIADEKNGGYLVGSTNYIFSVVTMNPAENPDFILYV

>JP_2x50

GTDGIITYEKDRLGNIVPGTEQVSQQTVDGKDVYTTISSTLQSFMETQMDAFLEK VKGKTATLVSAKTGEILATTQRPTFNADTKEGITEDFVWRDILYQSNYEPGSAMKVMTL ASSNNTFPSGEYFNSSEFKIADATTRDWDVNDGLTTGGMMTFLQGFAHSSNVGMSLLE QKMATWLDYLSRFKFGVPTRFGLTDEYAGQLPADNIVSIAQSSFGQGISVTQTQMLRAF TANDGVMLEPKFISAIYDTNNQSVRKSQKEIVGNPVSKEAASTTRNHMILVGTDPLYGT MHYTGKPIITVPGQNVAVKSGTAQIADEKNGGYLVGSTNYIFSVVTMNPAENPDFILYV

>JP_2x51

GTDGIITYEKDRLGNIVPGTEQVSQQTVDGKDVYTTISSTLQSFMETQMDAFQGK LKGKTATLVSAKTGEILATTQRPTFNADTKEGITEDFVWRDILYQSNYEPGSAMKVMTL ASSNNTFPSGEYFNSSEFKIADATTRDWDVNEGLTTGGMMTFLQGFAHSSNVGMSLLE QKMATWLDYLKRFKFGVPTRFGLTDEYAGQLPADNIVSIAQSSFGQGISVTQTQMLRAF TANDGVMLEPKFISAIYDTNNQSVRKSQKEIVGNPVSKEAASTTRNHMILVGTDPLYGT MHYTGKPIITVPGQNVAVKSGTAQIADEKNGGYLVGSTNYIFSVVTMNPAENPDFILYV

>JP_2x52

GTDGIITYEKDRLGNIVPGTEQVSQQTVDGKDVYTTISSPLQSFMETQMDAFQEK LKGKTATLVSAKTGEILATTQRPTFNADTKEGITEDFVWRDILYQSNYEPGSAFKVMML ASSNNTFPSGEYFNSSEFKIADATTRDWDVNEGLTTGGMMTFLQGFAHSSNVGTSLLEQ KMATWLDYLKRFKFGVPTRFGLTDEYAGQLPADNIVSIAQSSFGQGISVTQTQMLRAFT

ANDGVMLEPKFISAIYDTNNQSVRKSQKEIVGNPVSKEAASTTRNHMILVGTDPLYGTM
HYTGKPIITVPGQNVAVKSGTAQIADEKNGGYLVGSTNYIFSVVTMNPAENPDFILYV

>JP_2x53

GTDGIITYEKDRLGNIVPGTEQVSQRTMDGKDVYTTLSSPLQSFMETQMDAFLEK
VKGKTATLVSAKTGEILATTQRPTFNADTKEGITEDFVWRDILYQSNYEPGSAMKVMTL
ASSNNTFPSGEYFNSSEFKIADATTRDWDVNEGLTTGGMMTFLQGFAHSSNVGMSLLE
QKMATWLDYLKRFKFGVPTRFGLTDEYAGQLPADNIVSIAQSSFGQGISVTQTQMLRAF
TANDGVMLEPKFISAIYDTNNQSVRKSQKEIVGNPVSKEAASTTRNHMILVGTDPLYGT
MHYTGKPIITVPGQNVAVKSGTAQIADEKNGGYLVGSTNYIFSAVTMNPAENPDFILYV

>JP_2x54

GTDGIITYEKDRLGNIVPGTEQVSQRTMDGKDVYTTLSSPLQSFMETQMDAFLEK
VKGKTATLVSAKTGEILATTQRPTFNADTKEGITEDFVWRDILYQSNYEPGSAMKVMTL
ASSNNTFPSGEYFNSSEFKIADATTRDWDVNDGLTTGGMMTFLQGFAHSSNVGMSLLE
QKMATWLDYLKRFKFGVPTRFGLTDEYAGQLPADNIVSIAQSSFGQGISVTQTQMLRAF
TANDGVMLEPKFISAIYDTNNQSVRKSQKEIVGNPVSKEAASTTRNHMILVGTDPLYGT
MHYTGKPIITVPGQNVAVKSGTAQIADEKNGGYLVGSTNYIFSAVTMNPAENPDFILYV

>JP_2x55

GTDGIITYEKDRLGNIVPGTEQVSQRTMDGKDVYTTISSPLQSFMETQMDAFQEK
LKGKTATLVSAKTGEILATTQRPTFNADTKEGITEDFVWRDILYQSNYEPGSAMKVMTL
ASSNNTFPSGEYFNSSEFKIADATTRDWDVNAGLTTGGMMTFLQGFAHSSNVGMSLLE
QKMATWLDYLKRFKFGVPTRFGLTDEYAGQLPADNIVSIAQSSFGQGISVTQTQMLRAF
TANDGVMLEPKFISAIYDTNNQSVRKSQKEIVGNPVSKEAASTTRNHMILVGTDPLYGT
MHYTGKPIITVPGQNVAVKSGTAQIADEKNGGYLVGSTNYIFSAVTMNPAENPDFILYV

>JP_2x56

GTDGIITYEKDRLGNIVPGTEQVSQRTMDGKDVYTTISSPLQSFMETQMDAFQEK
VKGKTATLVSAKTGEILATTQRPTFDADTKEGITEDFVWRDILYQSNYEPGSTMKVMML
AAANNTFPGGEVFNSSELKIADATIRDWDVNEGLTVGGMMTFLQGFAHSSNVGMSLLE
QKMATWLDYLNRFKFGVPTRFGLTDEYAGQLPADNIVSIAQSSFGQGISVTQTQMLRAF

TANDGVMLEPKFISAIYDTNNQSVRKSQKEIVGKPVSEDAASLTRTNMILVGTDPLYGT
MHYTGKPIITVPGQNVAVKSGTAQIADEKNGGYLVGSTNYIFSVVTMNPAENPDFILYV

>JP_2x57

GKDGIITYEKDRLGNIVPGTEQVSQQTVDGKDVYTTLSSPLQSFMETQMDAFLEK
VKGKTATLVSAKTGEILATTQRPTFNADTKEGITEDFVWRDILYQSNYEPGSTMKVMTL
AAANNTFPGGEVFNSSELKIADVTIRDWDVNDGLTTGRMMTFLQGFALSSNVGMSLLE
QKMTTWLDYLNRFKFGVPTRFGLTDEYSGQLPEDNIVNIAQSSFGQGISVTQTQMLRAF
TANDGVMLEPKFISAIYDTNNQSVRKSQKEIVGKPVSEDAASLTRTNMILVGTDPLYGT
MHQTGKPIITVPGQNVAVKSGTAQIADEKNGGYLVGSTNYIFSVVTMNPAENPDFILYV

>JP_2x58

GTDGIITYEKDRLGNIVPGTEQVSQRTMDGKDVYTTISSPLQSFMETQMDAFQEK
VKGKTATLVSAKTGEILATTQRPTFDADTKEGITEDFVWRDILYQSNYEPGSAMKVMM
LAAANNTFPGGEVFNSSELKIADATIRDWDVNEGLTGGRMMTFSQGFAHSSNVGMTLL
EQKMATWLDYLNRFKFGVPTRFGLTDEYAGQLPADNIVNIAQSSFGQGISVTQTQMLR
AFTANDGVMLEPKFISALYDPNDQSVRKSQKEIVGNPVSKAAASSTREHMVMVGTDPV
YGTMHSTGKPNVNVPGQNVALKSGTAEIADEKNGGYLTGETNNIFSVVSMHPAENPDFI
LYV

>JP_2x59

GTDGIITYEKDRLGNIVPGTEQVSQRTMDGKDVYTTISSPLQSFMETQMDAFQEK
VKGKYMTATLVSAKTGEILATTQRPTFDADTKEGITEDFVWRDILYQSNYEPGSAMKV
MTLASSIDNNTFPSGEYFNSSEFKIADATTRDWDVNEGLTTGGMMTFLQGFAHSSNVG
MSLLEQKMGDATWLDYLKRFKFGVPTRFGLTDEYAGQLPADNIVNIAQSSFGQGISVTQ
TQMLRAFTAIANDGVMLEPKFISAIYDTNNQSVRKSQKEIVGNPVSKEAASTTRNHMIL
VGTDPLYGTMYNHYTGKPIITVPGQNVAVKSGTAQIADEKNGGYLVGSTNYIFSAVTM
NPAENPDFILYV

Supplementary References

1. Altschul SF, Gish W, Miller W, Myers EW, Lipman DJ. Basic local alignment search tool. J Mol Biol. 1990;215:403–10. PubMed https://doi.org/10.1016/S0022-2836(05)80360-2

2. Metcalf BJ, Gertz RE Jr, Gladstone RA, Walker H, Sherwood LK, Jackson D, et al.; Active Bacterial Core surveillance team. Strain features and distributions in pneumococci from children with invasive disease before and after 13-valent conjugate vaccine implementation in the USA. Clin Microbiol Infect. 2016;22:60.e9–29. PubMed https://doi.org/10.1016/j.cmi.2015.08.027

3. Nakano S, Fujisawa T, Ito Y, Chang B, Matsumura Y, Yamamoto M, et al. Spread of Meropenem-Resistant Streptococcus pneumoniae Serotype 15A-ST63 Clone in Japan, 2012-2014. Emerg Infect Dis. 2018;24:275–83. PubMed https://doi.org/10.3201/eid2402.171268

4. Nakano S, Fujisawa T, Ito Y, Chang B, Matsumura Y, Yamamoto M, et al. Whole-Genome Sequencing Analysis of Multidrug-Resistant Serotype 15A Streptococcus pneumoniae in Japan and the Emergence of a Highly Resistant Serotype 15A-ST9084 Clone. Antimicrob Agents Chemother. 2019;63:63. PubMed https://doi.org/10.1128/AAC.02579-18

5. Nakano S, Fujisawa T, Ito Y, Chang B, Matsumura Y, Yamamoto M, et al. Penicillin-Binding Protein Typing, Antibiotic Resistance Gene Identification, and Molecular Phylogenetic Analysis of Meropenem-Resistant Streptococcus pneumoniae Serotype 19A-CC3111 Strains in Japan. Antimicrob Agents Chemother. 2019;63:63. PubMed https://doi.org/10.1128/AAC.00711-19

6. Croucher NJ, Page AJ, Connor TR, Delaney AJ, Keane JA, Bentley SD, et al. Rapid phylogenetic analysis of large samples of recombinant bacterial whole genome sequences using Gubbins. Nucleic Acids Res. 2015;43:e15. PubMed https://doi.org/10.1093/nar/gku1196

7. Chen S, Zhou Y, Chen Y, Gu J. fastp: an ultra-fast all-in-one FASTQ preprocessor. Bioinformatics. 2018;34:i884–90. PubMed https://doi.org/10.1093/bioinformatics/bty560

8. Li H, Durbin R. Fast and accurate short read alignment with Burrows-Wheeler transform. Bioinformatics. 2009;25:1754–60. PubMed https://doi.org/10.1093/bioinformatics/btp324

9. McKenna A, Hanna M, Banks E, Sivachenko A, Cibulskis K, Kernytsky A, et al. The Genome Analysis Toolkit: a MapReduce framework for analyzing next-generation DNA sequencing data. Genome Res. 2010;20:1297–303. PubMed https://doi.org/10.1101/gr.107524.110

10. Danecek P, Auton A, Abecasis G, Albers CA, Banks E, DePristo MA, et al.; 1000 Genomes Project Analysis Group. The variant call format and VCFtools. Bioinformatics. 2011;27:2156–8. PubMed https://doi.org/10.1093/bioinformatics/btr330

11. Stamatakis A. RAxML version 8: a tool for phylogenetic analysis and post-analysis of large phylogenies. Bioinformatics. 2014;30:1312–3. PubMed https://doi.org/10.1093/bioinformatics/btu033

12. Seemann T. Prokka: rapid prokaryotic genome annotation. Bioinformatics. 2014;30:2068–9. PubMed https://doi.org/10.1093/bioinformatics/btu153

13. Carver T, Berriman M, Tivey A, Patel C, Böhme U, Barrell BG, et al. Artemis and ACT: viewing, annotating and comparing sequences stored in a relational database. Bioinformatics. 2008;24:2672–6. PubMed https://doi.org/10.1093/bioinformatics/btn529

14. Kozlov AM, Darriba D, Flouri T, Morel B, Stamatakis A. RAxML-NG: a fast, scalable and user-friendly tool for maximum likelihood phylogenetic inference. Bioinformatics. 2019;35:4453–5. PubMed https://doi.org/10.1093/bioinformatics/btz305

15. Page AJ, Cummins CA, Hunt M, Wong VK, Reuter S, Holden MT, et al. Roary: rapid large-scale prokaryote pan genome analysis. Bioinformatics. 2015;31:3691–3. PubMed https://doi.org/10.1093/bioinformatics/btv421

16. Rambaut A, Lam TT, Max Carvalho L, Pybus OG. Exploring the temporal structure of heterochronous sequences using TempEst (formerly Path-O-Gen). Virus Evol. 2016;2:vew007. PubMed https://doi.org/10.1093/ve/vew007

17. Lemey P, Rambaut A, Drummond AJ, Suchard MA. Bayesian phylogeography finds its roots. PLOS Comput Biol. 2009;5:e1000520. PubMed https://doi.org/10.1371/journal.pcbi.1000520

Appendix Table 1. Strain information of the isolates tested in this study

					PBP profile			MIC (µg/ml)*				
Isolate name	Accession No.	Serotype	Sequence type	Region	*pbp1a*	*pbp2b*	*pbp2x*	PCG	CTX	MEM	EM	LFX
PC0217	DRR198414	12F	4846	Tokai	37	JP14	JP27	0.12	≤0.06	≤0.06	>128	1
PC0786	DRR198415	12F	4846	Kinki	37	JP14	JP27	0.12	≤0.06	≤0.06	>128	1
PC0822	DRR198416	12F	4846	Kanto	37	JP14	JP27	≤0.06	≤0.06	≤0.06	>128	1
PC0827	DRR198417	12F	4846	Kinki	37	JP14	JP27	0.12	≤0.06	≤0.06	>128	1
PC0872	DRR198418	12F	4846	Kanto	37	JP14	JP27	0.12	≤0.06	≤0.06	>128	1
PC0882	DRR198419	12F	4846	Kanto	37	JP14	JP27	≤0.06	≤0.06	≤0.06	>128	1
PC0896	DRR198420	12F	4846	Kanto	37	JP14	JP27	≤0.06	≤0.06	≤0.06	>128	1
PC0974	DRR198421	12F	4846	Tokai	37	JP14	JP27	0.12	≤0.06	≤0.06	>128	1
PC1034	DRR198422	12F	4846	Kanto	37	JP14	JP23	0.12	0.5	≤0.06	>128	1
PC1035	DRR198423	12F	6945	Kinki	37	4	23	≤0.06	≤0.06	≤0.06	>128	1
PC1037	DRR198424	12F	4846	Kyusyu	37	JP14	JP27	0.12	≤0.06	≤0.06	>128	1
PC1050	DRR198425	12F	6945	Kinki	37	4	23	≤0.06	≤0.06	≤0.06	>128	1
PC1051	DRR198426	12F	4846	Tohoku	37	JP14	JP27	0.12	≤0.06	≤0.06	>128	1
PC1053	DRR198427	12F	4846	Kinki	37	JP14	JP27	0.12	≤0.06	≤0.06	>128	1
PC1084	DRR198428	12F	4846	Kanto	37	JP14	JP27	0.12	≤0.06	≤0.06	>128	1
PC1114	DRR198429	12F	4846	Tohoku	37	JP14	JP27	0.12	≤0.06	≤0.06	>128	1
PC1150	DRR198430	12F	6945	Tokai	37	4	23	≤0.06	≤0.06	≤0.06	>128	1
PC1182	DRR198431	12F	4846	Tokai	37	JP14	JP27	0.12	≤0.06	≤0.06	>128	1
PC1185	DRR198432	12F	4846	Kanto	37	JP14	JP27	0.12	≤0.06	≤0.06	>128	1
PC1198	DRR198433	12F	4846	Tohoku	37	JP14	JP27	0.12	≤0.06	≤0.06	>128	1
PC1203	DRR198434	12F	4846	Kanto	37	JP14	JP27	0.12	≤0.06	≤0.06	>128	1
PC1266	DRR198435	12F	4846	Kinki	37	JP14	JP27	≤0.06	≤0.06	≤0.06	>128	1
PC1271	DRR198436	12F	4846	Kanto	37	JP14	JP27	0.12	≤0.06	≤0.06	>128	1
PC1277	DRR198437	12F	4846	Kanto	37	JP14	JP27	≤0.06	≤0.06	≤0.06	>128	1
PC1278	DRR198438	12F	4846	Tohoku	37	JP14	JP27	0.12	≤0.06	≤0.06	>128	1
PC1283	DRR198439	12F	4846	Tohoku	37	JP14	JP27	0.12	≤0.06	≤0.06	>128	1
PC1285	DRR198440	12F	4846	Kinki	37	JP14	JP27	0.12	0.12	≤0.06	>128	1
PC1286	DRR198441	12F	4846	Kanto	37	JP14	JP27	0.12	≤0.06	≤0.06	>128	1
PC1287	DRR198442	12F	4846	Sanin	37	JP14	JP27	≤0.06	≤0.06	≤0.06	>128	1
PC1293	DRR198443	12F	4846	Sanin	37	JP14	JP27	0.12	≤0.06	≤0.06	>128	1
PC1296	DRR198444	12F	4846	Tokai	37	JP14	JP27	≤0.06	≤0.06	≤0.06	>128	1
PC1305	DRR198445	12F	4846	Tokai	37	JP14	JP27	0.12	0.12	≤0.06	>128	1
PC1314	DRR198446	12F	4846	Tokai	37	JP14	JP27	0.12	≤0.06	≤0.06	>128	0.5
PC1347	DRR198447	12F	4846	Tokai	37	JP14	JP27	0.12	≤0.06	≤0.06	>128	1
PC1350	DRR198448	12F	4846	Kanto	37	JP14	JP27	≤0.06	≤0.06	≤0.06	>128	1
PC1355	DRR198449	12F	4846	Tokai	37	JP14	JP27	≤0.06	≤0.06	≤0.06	>128	0.5
PC1356	DRR198450	12F	4846	Kanto	37	JP14	JP27	≤0.06	≤0.06	≤0.06	>128	0.5
PC1364	DRR198451	12F	4846	Tokai	37	JP14	JP27	≤0.06	≤0.06	≤0.06	>128	1
PC1371	DRR198452	12F	6945	Kinki	37	4	23	≤0.06	≤0.06	≤0.06	>128	1
PC1376	DRR198453	12F	4846	Tohoku	37	JP14	JP27	≤0.06	≤0.06	≤0.06	>128	1
PC1382	DRR198454	12F	6945	Tokai	37	4	23	≤0.06	≤0.06	≤0.06	>128	1
PC1384	DRR198455	12F	6945	Sanin	37	4	23	0.12	≤0.06	≤0.06	≤0.06	1
PC1385	DRR198456	12F	4846	Kinki	37	JP14	JP27	0.12	≤0.06	≤0.06	>128	1
PC1386	DRR198457	12F	4846	Kanto	37	JP14	JP27	0.12	≤0.06	≤0.06	>128	1
PC1387	DRR198458	12F	6945	Kinki	37	4	23	≤0.06	≤0.06	≤0.06	>128	1
PC1389	DRR198459	12F	4846	Kyusyu	37	JP14	JP27	0.12	≤0.06	≤0.06	>128	1
PC1390	DRR198460	12F	4846	Kanto	37	JP14	JP27	0.12	≤0.06	≤0.06	>128	1
PC1394	DRR198461	12F	4846	Sanin	37	JP14	JP27	≤0.06	≤0.06	≤0.06	>128	1
PC1395	DRR198462	12F	4846	Kanto	37	JP14	JP27	0.12	≤0.06	≤0.06	>128	1
PC1397	DRR198463	12F	6945	Kinki	37	4	23	≤0.06	≤0.06	≤0.06	>128	1
PC1399	DRR198464	12F	6945	Kinki	37	4	23	≤0.06	≤0.06	≤0.06	>128	1
PC1403	DRR198465	12F	4846	Kinki	37	JP14	JP27	0.12	0.12	≤0.06	>128	1
PC1407	DRR198466	12F	6945	Sanin	37	4	23	≤0.06	≤0.06	≤0.06	≤0.06	1
PC1410	DRR198467	12F	6945	Kinki	37	4	23	≤0.06	≤0.06	≤0.06	>128	1
PC1412	DRR198468	12F	4846	Tohoku	37	JP14	JP27	0.12	≤0.06	≤0.06	>128	1
PC1416	DRR198469	12F	4846	Sanin	37	JP14	JP27	0.12	≤0.06	≤0.06	>128	1
PC1426	DRR198470	12F	4846	Tokai	37	JP14	JP27	0.12	≤0.06	≤0.06	>128	1
PC1429	DRR198471	12F	6945	Kinki	37	4	23	≤0.06	≤0.06	≤0.06	>128	1
PC1438	DRR198472	12F	4846	Tokai	37	JP14	JP27	0.12	≤0.06	≤0.06	>128	1
PC1442	DRR198473	12F	4846	Tohoku	37	JP14	JP27	0.12	≤0.06	≤0.06	>128	1
PC1450	DRR198474	12F	4846	Kyusyu	37	JP14	JP27	0.12	≤0.06	≤0.06	>128	1
PC1465	DRR198475	12F	4846	Tohoku	37	JP14	JP27	0.25	≤0.06	≤0.06	>128	1
PC1467	DRR198476	12F	4846	Kyusyu	37	JP14	JP27	0.12	≤0.06	≤0.06	>128	1
PC1499	DRR198477	12F	6945	Kinki	37	4	23	≤0.06	≤0.06	≤0.06	>128	1
PC1532	DRR198479	12F	4846	Tokai	37	JP14	JP58	0.12	≤0.06	≤0.06	>128	1

					PBP profile			MIC (µg/ml)*				
Isolate name	Accession No.	Serotype	Sequence type	Region	*pbp1a*	*pbp2b*	*pbp2x*	PCG	CTX	MEM	EM	LFX
PC1536	DRR198480	12F	6945	Kyusyu	37	4	23	≤0.06	≤0.06	≤0.06	≤0.06	1
PC1538	DRR198481	12F	4846	Tohoku	37	JP14	JP27	0.12	≤0.06	≤0.06	>128	1
PC1540	DRR198482	12F	4846	Kinki	37	JP14	JP59	0.12	0.5	≤0.06	>128	1
PC1544	DRR198483	12F	4846	Kyusyu	37	JP14	JP27	0.12	≤0.06	≤0.06	>128	1
PC1554	DRR198484	12F	4846	Kyusyu	37	JP14	JP27	0.12	≤0.06	≤0.06	>128	1
PC1557	DRR198485	12F	4846	Tokai	37	JP14	JP27	≤0.06	≤0.06	≤0.06	>128	1
PC1565	DRR198486	12F	6945	Kinki	37	4	23	≤0.06	≤0.06	≤0.06	>128	1
PC1567	DRR198487	12F	4846	Kanto	37	JP14	JP27	≤0.06	≤0.06	≤0.06	>128	1
PC1568	DRR198488	12F	6945	Kinki	37	4	23	≤0.06	≤0.06	≤0.06	>128	1

*PCG, penicillin; CTX, cefotaxime; MEM, meropenem; EM, erythromycin; LFX, levofloxacin.

Appendix Table 2. Mapping coverage and genetic characteristics of the isolates tested in this study.

									folA mutation					
Isolate name	Coverage	N_{50}	N_{70}	*ermB*	*ermTR*	*mef*	Pili1	Pili2	I100L	D92R	*folP*_insertion	*tetM*	*tetO*	*tet*(S/M)
PC0217	97.7461	64385	45761	Yes	No	No	No	No	Yes	No	Yes	Yes	No	No
PC0786	78.5059	41865	25372	Yes	No	No	No	No	Yes	No	Yes	Yes	No	No
PC0822	78.8616	52525	28169	Yes	No	No	No	No	No	No	Yes	Yes	No	No
PC0827	81.6176	56700	31674	Yes	No	No	No	No	No	No	Yes	Yes	No	No
PC0872	66.3265	40005	21885	Yes	No	No	No	No	No	No	Yes	Yes	No	No
PC0882	71.4055	57474	31674	Yes	No	No	No	No	No	No	Yes	Yes	No	No
PC0896	158.557	60391	34894	Yes	No	No	No	No	No	No	Yes	Yes	No	No
PC0974	68.1011	55241	22704	Yes	No	No	No	No	Yes	No	Yes	Yes	No	No
PC1034	126.533	60391	34921	Yes	No	No	No	No	No	No	Yes	Yes	No	No
PC1035	94.4535	63775	33872	Yes	No	No	No	No	No	No	No	Yes	No	No
PC1037	88.866	55241	30257	Yes	No	No	No	No	Yes	No	Yes	Yes	No	No
PC1050	76.4846	18220	10453	Yes	No	No	No	No	No	No	No	Yes	No	No
PC1051	167.889	62638	42644	Yes	No	No	No	No	Yes	No	Yes	Yes	No	No
PC1053	107.877	63628	36760	Yes	No	No	No	No	Yes	No	Yes	Yes	No	No
PC1084	150.26	45969	25243	Yes	No	No	No	No	Yes	No	Yes	Yes	No	No
PC1114	45.3837	40266	20946	Yes	No	No	No	No	Yes	No	Yes	Yes	No	No
PC1150	61.9118	58103	31855	Yes	No	No	No	No	No	No	No	Yes	No	No
PC1182	129.796	62638	39115	Yes	No	No	No	No	Yes	No	Yes	Yes	No	No
PC1185	60.9356	58492	26882	Yes	No	No	No	No	Yes	No	Yes	Yes	No	No
PC1198	41.6472	51813	26882	Yes	No	No	No	No	Yes	No	Yes	Yes	No	No
PC1203	51.6374	55274	23990	Yes	No	No	No	No	Yes	No	Yes	Yes	No	No
PC1266	181.194	51362	26881	Yes	No	No	No	No	Yes	No	Yes	Yes	No	No
PC1271	70.983	64206	33804	Yes	No	No	No	No	Yes	No	Yes	Yes	No	No
PC1277	136.951	55241	26397	Yes	No	No	No	No	Yes	No	Yes	Yes	No	No
PC1278	102.953	45101	24472	Yes	No	No	No	No	Yes	No	Yes	Yes	No	No
PC1283	133.917	60391	34921	Yes	No	No	No	No	Yes	No	Yes	Yes	No	No
PC1285	188.722	59453	43996	Yes	No	No	No	No	Yes	No	Yes	Yes	No	No
PC1286	93.991	72107	42926	Yes	No	No	No	No	Yes	No	Yes	Yes	No	No
PC1287	77.8085	59498	42926	Yes	No	No	No	No	Yes	No	Yes	Yes	No	No
PC1293	148.198	57257	34280	Yes	No	No	No	No	Yes	No	Yes	Yes	No	No
PC1296	66.2212	60385	34921	Yes	No	No	No	No	Yes	No	Yes	Yes	No	No
PC1305	112.115	62638	39352	Yes	No	No	No	No	Yes	No	Yes	Yes	No	No
PC1314	151.22	48407	28569	Yes	No	No	No	No	Yes	No	Yes	Yes	No	No
PC1347	103.943	58827	37231	Yes	No	No	No	No	Yes	No	Yes	Yes	No	No
PC1350	84.8507	58836	33812	Yes	No	No	No	No	Yes	No	Yes	Yes	No	No
PC1355	85.4997	14795	8189	Yes	No	No	No	No	Yes	No	Yes	Yes	No	No
PC1356	72.7795	60395	41736	Yes	No	No	No	No	Yes	No	Yes	Yes	No	No
PC1364	87.9509	70612	51434	Yes	No	No	No	No	Yes	No	Yes	Yes	No	No
PC1371	77.9551	45103	23126	Yes	No	No	No	No	No	No	No	Yes	No	No
PC1376	75.953	65767	45623	Yes	No	No	No	No	Yes	No	Yes	Yes	No	No
PC1382	111.501	56760	26157	Yes	No	No	No	No	No	No	No	Yes	No	No
PC1384	87.6827	48436	24384	No	No	No	No	No	No	No	No	Yes	No	No
PC1385	93.9111	52230	28116	Yes	No	No	No	No	Yes	No	Yes	Yes	No	No
PC1386	111.589	54898	23990	Yes	No	No	No	No	Yes	No	Yes	Yes	No	No
PC1387	95.3195	51531	24460	Yes	No	No	No	No	No	No	No	Yes	No	No
PC1389	91.3471	57257	25061	Yes	No	No	No	No	Yes	No	Yes	Yes	No	No
PC1390	54.8823	34473	18090	Yes	No	No	No	No	Yes	No	Yes	Yes	No	No
PC1394	103.749	59543	34948	Yes	No	No	No	No	Yes	No	Yes	Yes	No	No
PC1395	73.0218	59498	28213	Yes	No	No	No	No	Yes	No	Yes	Yes	No	No
PC1397	101.411	60389	31592	Yes	No	No	No	No	No	No	No	Yes	No	No
PC1399	111.571	62636	33870	Yes	No	No	No	No	No	No	No	Yes	No	No
PC1403	93.9766	46763	25061	Yes	No	No	No	No	Yes	No	Yes	Yes	No	No

Isolate name	Coverage	N_{50}	N_{70}	*ermB*	*ermTR*	*mef*	Pili1	Pili2	*folA* mutation I100L	*folA* mutation D92R	*folP*_insertion	*tetM*	*tetO*	*tet*(S/M)
PC1407	82.8333	45091	24010	No	No	No	No	No	No	No	No	Yes	No	No
PC1410	103.535	63775	33857	Yes	No	No	No	No	No	No	No	Yes	No	No
PC1412	88.8179	60388	31674	Yes	No	No	No	No	Yes	No	Yes	Yes	No	No
PC1416	146.909	54108	26882	Yes	No	No	No	No	Yes	No	Yes	Yes	No	No
PC1426	107.283	45091	23950	Yes	No	No	No	No	Yes	No	Yes	Yes	No	No
PC1429	137.678	54628	25068	Yes	No	No	No	No	No	No	No	Yes	No	No
PC1438	136.557	41446	20566	Yes	No	No	No	No	Yes	No	Yes	Yes	No	No
PC1442	140.574	55211	24257	Yes	No	No	No	No	Yes	No	Yes	Yes	No	No
PC1450	145.005	61471	34081	Yes	No	No	No	No	Yes	No	Yes	Yes	No	No
PC1465	151.159	65767	34871	Yes	No	No	No	No	Yes	No	Yes	Yes	No	No
PC1467	104.569	51362	24011	Yes	No	No	No	No	Yes	No	Yes	Yes	No	No
PC1499	128.857	63775	34488	Yes	No	No	No	No	NA	NA	No	Yes	No	No
PC1532	217.819	56132	30642	Yes	No	No	No	No	Yes	No	Yes	Yes	No	No
PC1536	75.7587	59643	28863	No	No	No	No	No	No	No	No	Yes	No	No
PC1538	89.3087	60942	42997	Yes	No	No	No	No	Yes	No	Yes	Yes	No	No
PC1540	95.9846	108974	63109	Yes	No	No	No	No	Yes	No	Yes	Yes	No	No
PC1544	184.055	97606	69764	Yes	No	No	No	No	Yes	No	Yes	Yes	No	No
PC1554	112.844	122409	60696	Yes	No	No	No	No	Yes	No	Yes	Yes	No	No
PC1557	108.202	87034	53299	Yes	No	No	No	No	Yes	No	Yes	Yes	No	No
PC1565	107.677	85128	48044	Yes	No	No	No	No	No	No	No	Yes	No	No
PC1567	135.756	93700	60031	Yes	No	No	No	No	Yes	No	Yes	Yes	No	No
PC1568	162.995	90146	65028	Yes	No	No	No	No	No	No	No	Yes	No	No

Appendix Table 3. Five genes that were identified exclusively in PC-JP12F isolates

Protein name	Sequence ID
Uncharacterized protein, Uncharacterized protein, NTP pyrophosphohydrolase including oxidative damage repair enzymes	CKV79152.1, CKW99395.1, VKN64777.1
Uncharacterized protein, Uncharacterized protein, Uncharacterized protein, Uncharacterized protein, Uncharacterized protein	CIS34299.1, CIT10688.1, CIU07372.1, CIV31640.1, CIZ29293.1,
Hypothetical protein	BBG82149.1
Hypothetical protein SP2UMMC_09170	KAA00665.1
Putative membrane protein, Membrane protein, Membrane protein, Uncharacterized protein, Uncharacterized protein	EJG79121.1, KAA03079.1, KAA03160.1, CEO70970.1, CEV43084.1

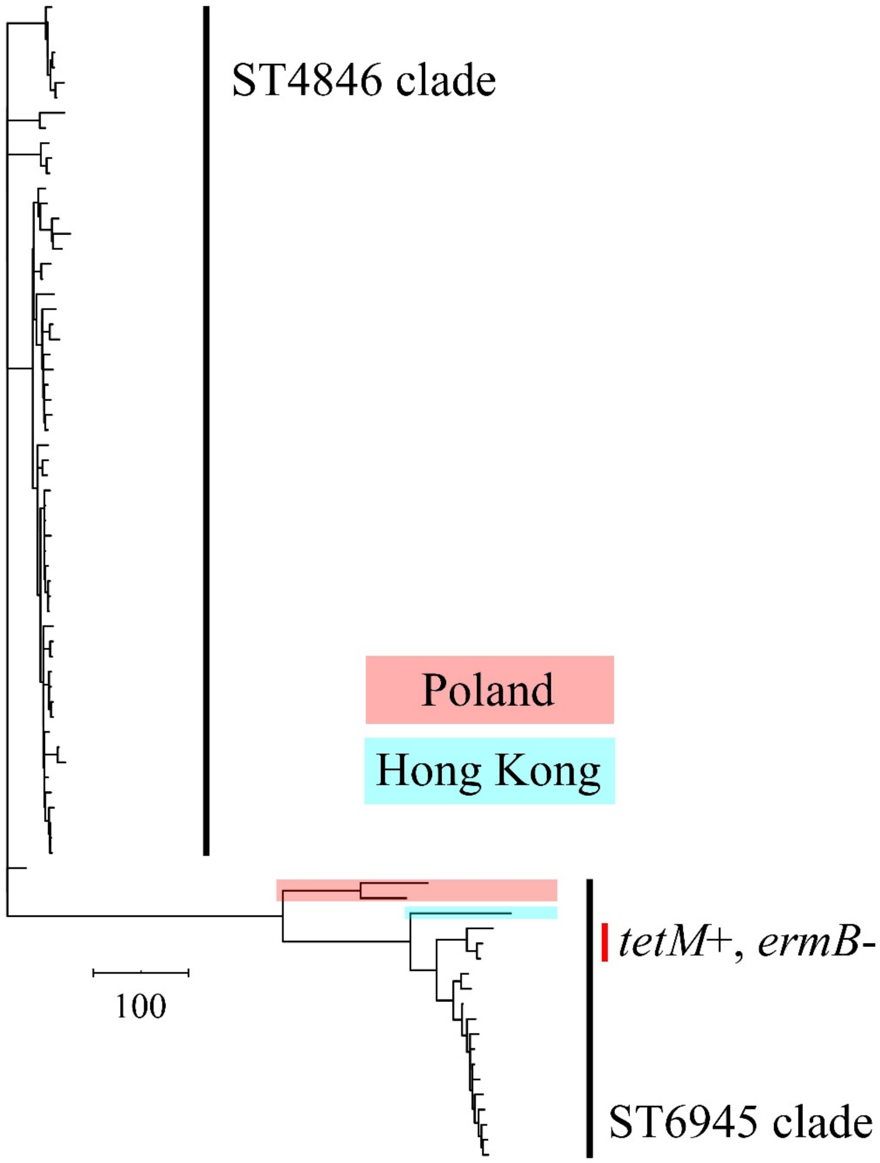

Appendix Figure 1. Recombination-free maximum-likelihood tree of serotype 12F-CC4846 isolates and three GPCS334 isolates from Poland and Hong Kong. All three isolates were clustered into an ST6945 clade. Two isolates from Poland carried neither *tetM* nor *ermB*. Three isolates in a ST6945 clade from Japan, marked with a red line, did not carry *ermB*.

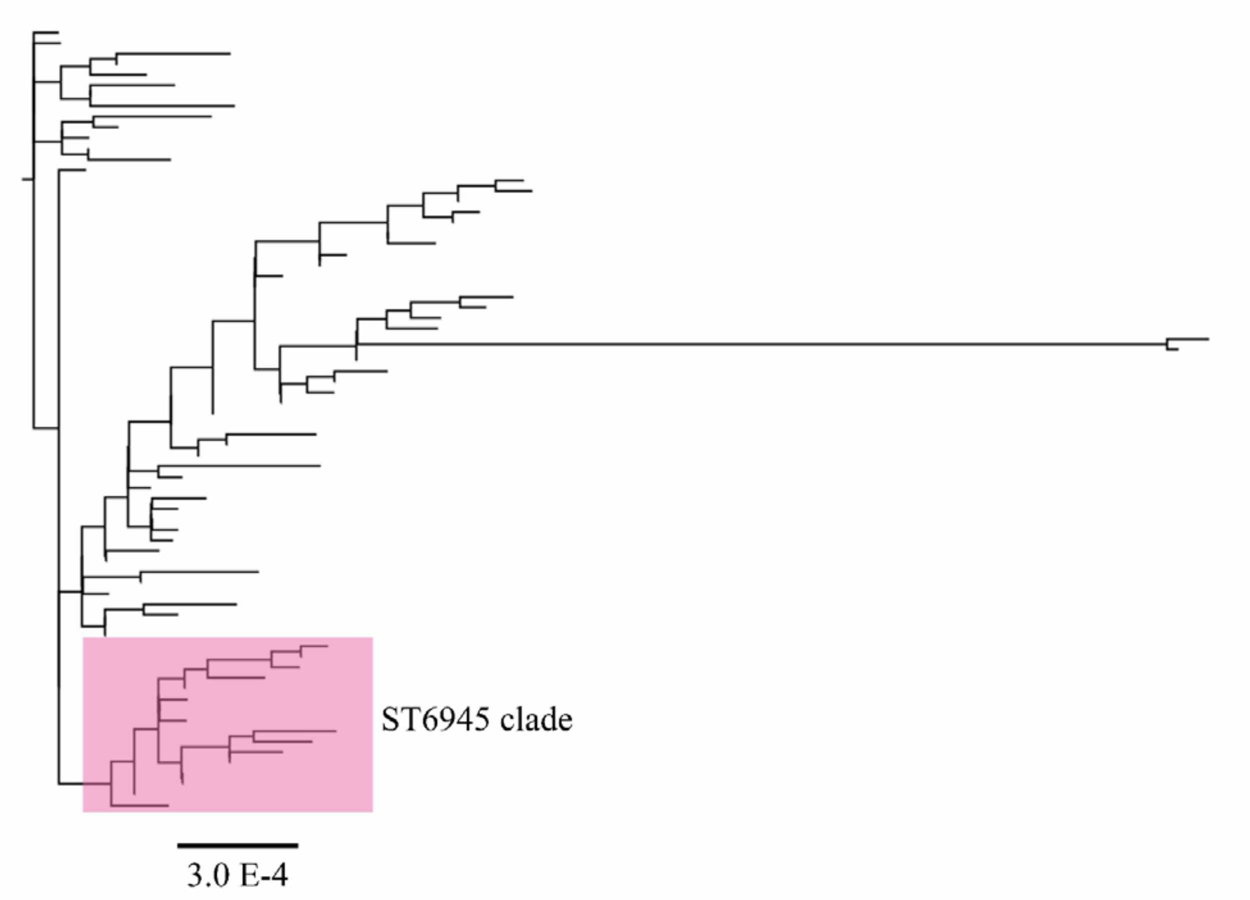

Appendix Figure 2. Maximum likelihood phylogenetic tree of serotype 12F *cps* locus sequences. The ST6945 isolates were exclusively included in the same cluster.

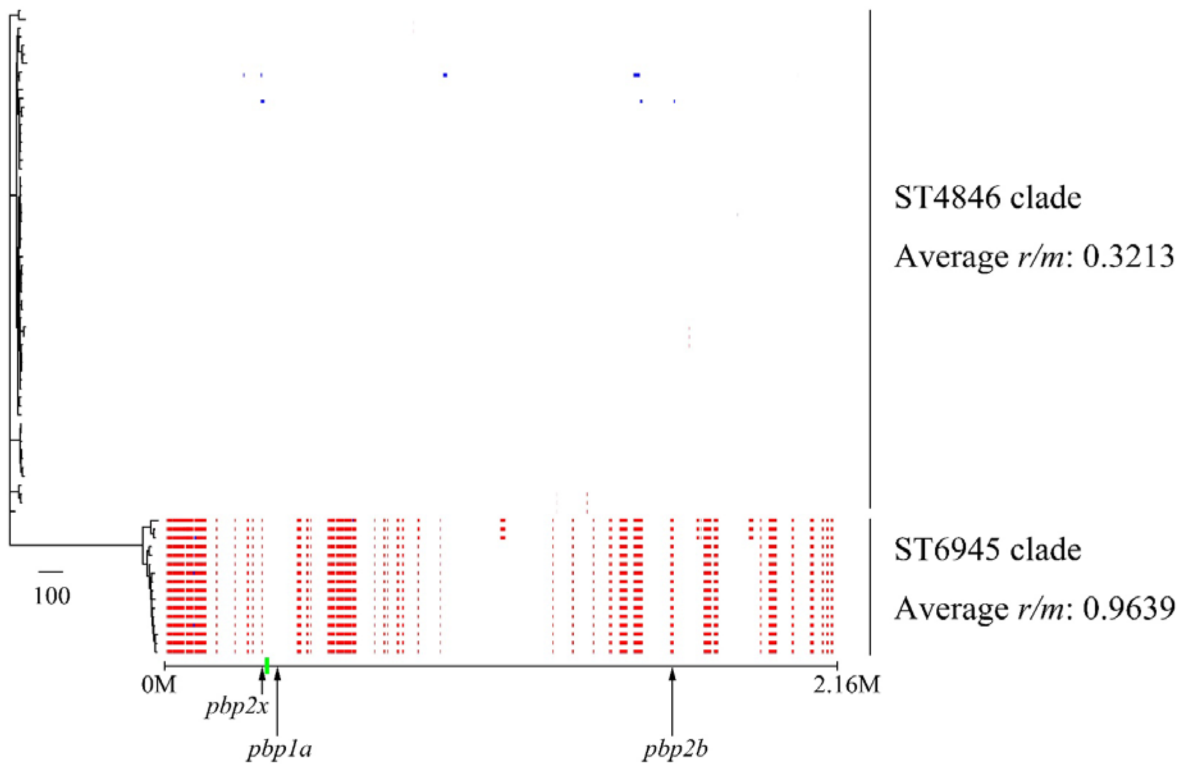

Appendix Figure 3. Phylogenetic tree and predicted recombination sites generated with Gubbins for serotype 12F isolates recovered in Japan. The blue blocks are unique to a single isolate, whereas the red blocks are shared by multiple isolates. The horizontal position of the blocks represents their position in the reference *S. pneumoniae* ASP0581 sequence (GenBank accession No. NZ_AP019192.2). The light green box on the coordinate line shows the *cps* locus of *S. pneumoniae* ASP0581 (GenBank accession No. NZ_AP019192.2), which was used as the reference sequence for mapping.

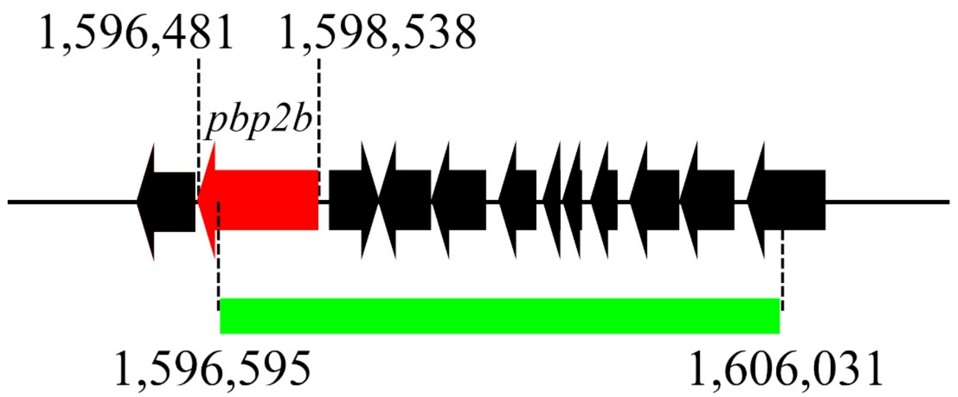

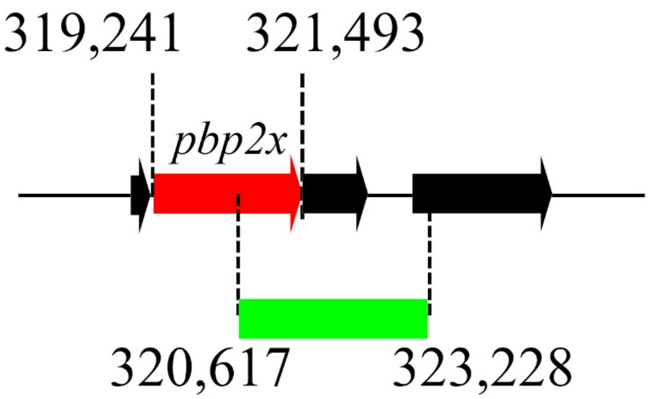

Appendix Figure 4. Sketch of the predicted recombination sites, including *pbp2b* and *pbp2x*. The light green blocks show the recombination sites. Each number shows the sequence coordinates based on *S. pneumoniae* ASP0581 (GenBank accession No. NZ_AP019192.2). These two recombination sites were shared by all of the ST6945 isolates.

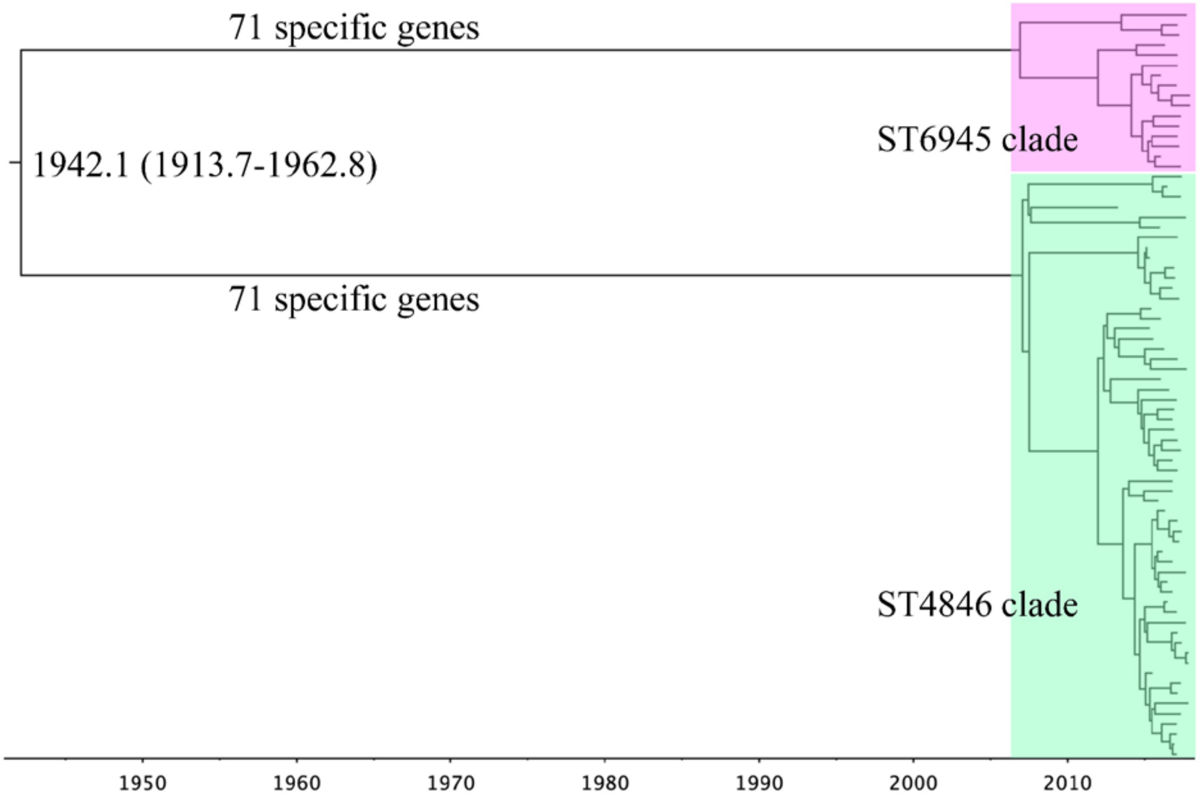

Appendix Figure 5. Maximum clade credibility tree of serotype 12F-CC4846 isolates in Japan created by BEAST. The time of the most recent common ancestor (TMRCA) is shown on the tree with 95% HPD.

The ST6945 and ST4846 isolates included in the clades are highlighted in pink and light green, respectively.

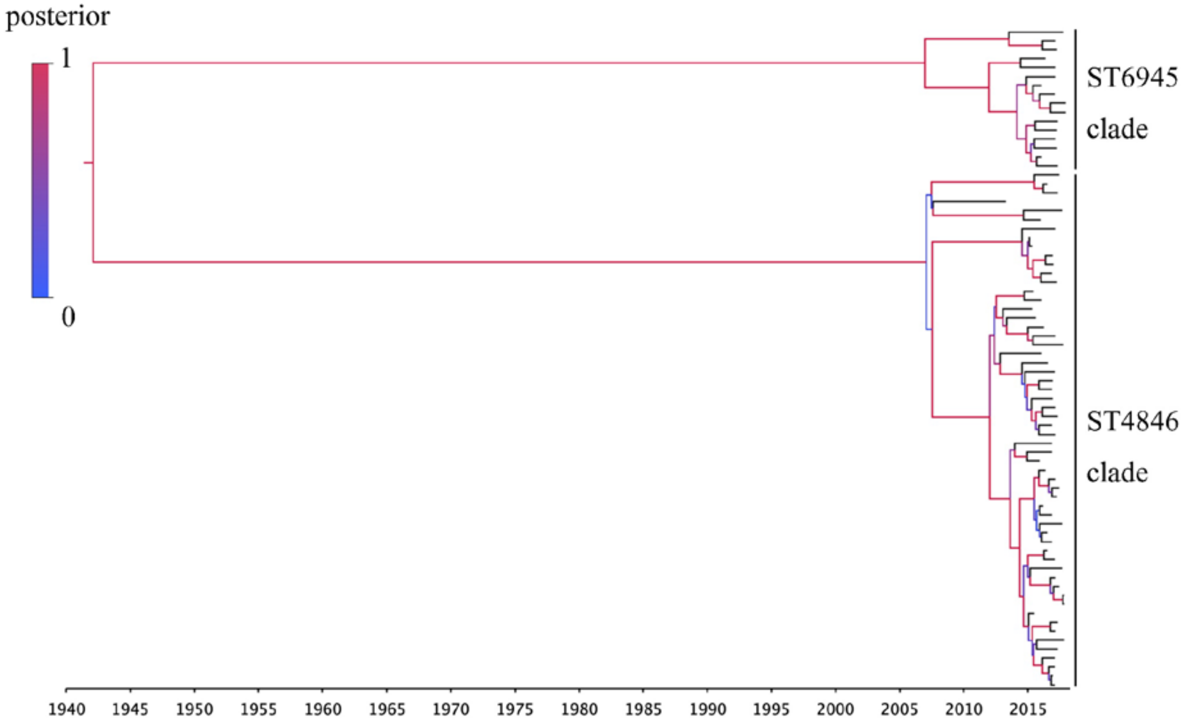

Appendix Figure 6. Maximum clade credibility tree of the serotype 12F-CC4846 isolates recovered in Japan created by BEAST. The posterior probabilities for each branch are included.

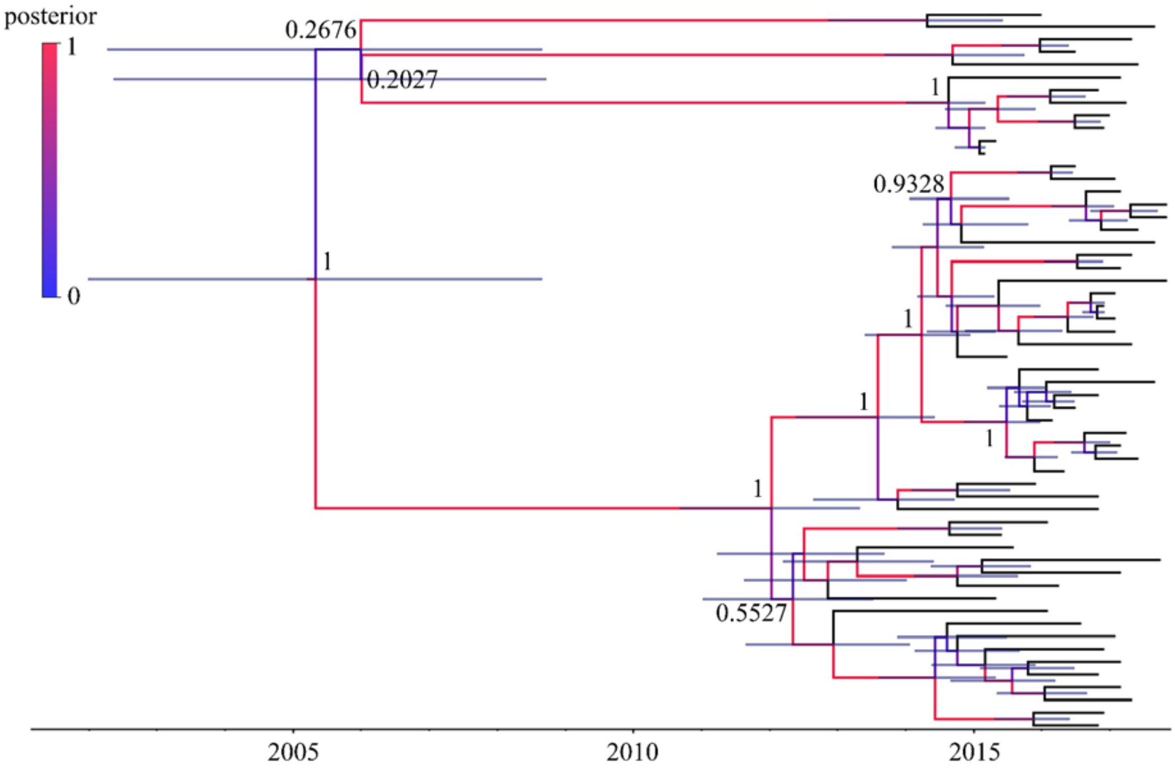

Appendix Figure 7. Maximum clade credibility tree of ST4846 clade isolates created by BEAST. The posterior probabilities of each branch are shown. The blue bars on each node indicate 95% HPD of the times of the most recent common ancestor (TMRCAs). The numbers on the branches indicate the posterior probabilities.

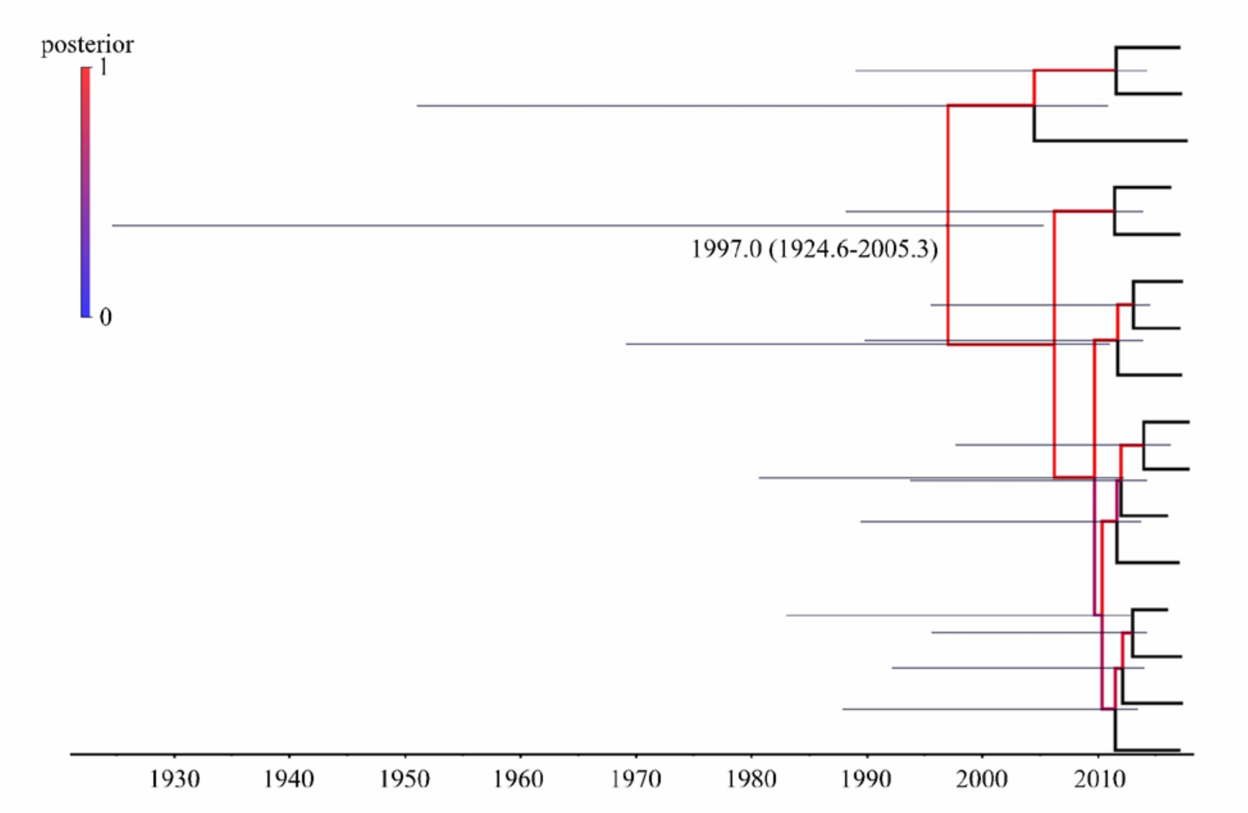

Appendix Figure 8. Maximum clade credibility tree of the ST6945 clade isolates created by BEAST. The posterior probabilities of each branch are shown. The time of the most recent common ancestor (TMRCA) is shown on the tree with 95% HPD. The blue bars on each node indicate 95% HPD of the TMRCAs.

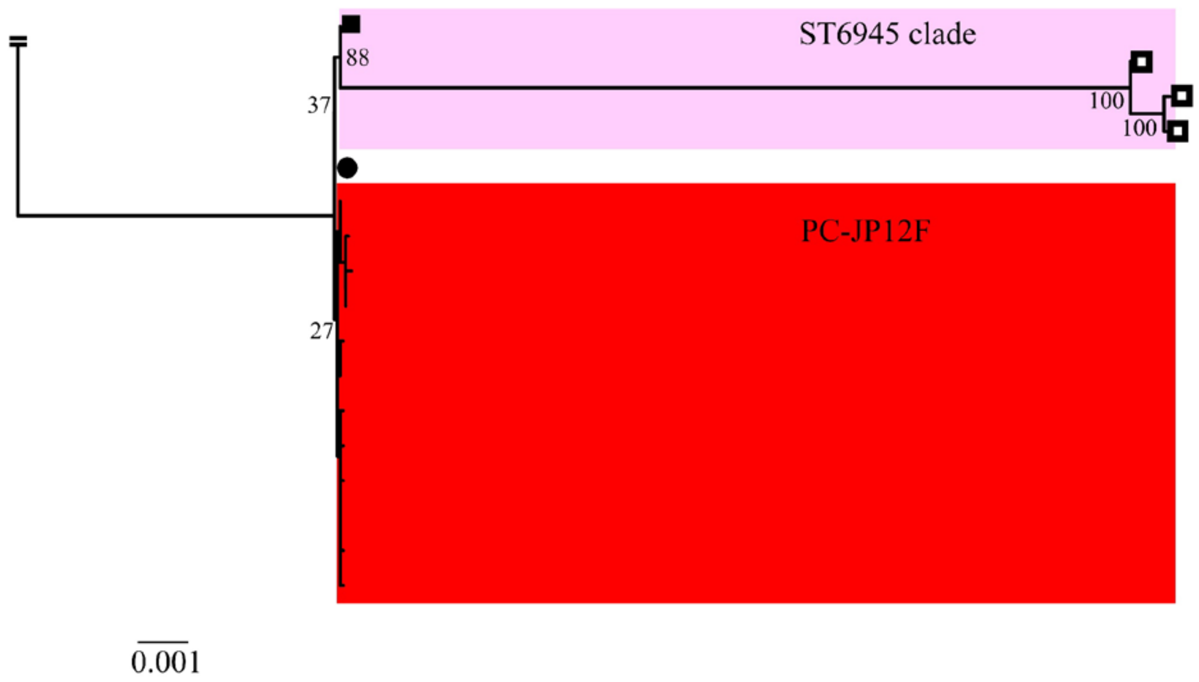

Appendix Figure 9. Phylogenetic tree of the *Tn*916 region of serotype 12F-CC4846 isolates recovered in Japan. The reference sequence of *Tn*916 (GenBank accession No. U09422.1) was used for the outgroup. All the isolates shown in pink are ST6945 isolates, and all the isolates shown in red are PC-JP12F isolates. A black square includes all 13 serotype 12F-ST6945 isolates with *ermB*, and each white square indicates a 12F-ST6945 isolate without *ermB*. A black circle indicates all 13 serotype 12F-ST4846 isolates except PC-JP12F isolates. The numbers on branches indicate bootstrap values.
